# Impact of RSVpreF vaccination on reducing the burden of respiratory syncytial virus in infants and older adults

**DOI:** 10.1038/s41591-024-03431-7

**Published:** 2025-01-09

**Authors:** Zhanwei Du, Abhishek Pandey, Seyed M. Moghadas, Yuan Bai, Lin Wang, Laura Matrajt, Burton H. Singer, Alison P. Galvani

**Affiliations:** 1 WHO Collaborating Center for Infectious Disease Epidemiology and Control, School of Public Health, LKS Faculty of Medicine, The University of Hong Kong, Hong Kong Special Administrative Region, Hong Kong, China; 2 School of Medicine, Yunnan University, Kunming, Yunnan China; 3 Laboratory of Data Discovery for Health Limited, Hong Kong Science Park, Hong Kong Special Administrative Region, China; 4 Center for Infectious Disease Modeling and Analysis, Yale School of Public Health, New Haven, CT USA; 5 Division of Communicable Disease Control and Prevention, Shenzhen Center for Disease Control and Prevention, Shenzhen, China; 6 Public Health Emergency Management Innovation Center, Beijing, China; 7 Agent-Based Modelling Laboratory, York University, Toronto, Ontario Canada; 8 Department of Genetics, University of Cambridge, Cambridge, UK; 9 Vaccine and Infectious Disease Division, Fred Hutchinson Cancer Center, Seattle, WA USA; 10 Emerging Pathogens Institute, University of Florida, Gainesville, FL USA

**Keywords:** Infectious diseases, Medical research

## Abstract

Respiratory syncytial virus (RSV) causes a substantial health burden among infants and older adults. Prefusion F protein-based vaccines have shown high efficacy against RSV disease in clinical trials, offering promise for mitigating this burden through maternal and older adult immunization. Employing an individual-based model, we evaluated the impact of RSV vaccination on hospitalizations and deaths in 13 high-income countries, assuming that the vaccine does not prevent infection or transmission. Using country-specific vaccine uptake rates for seasonal influenza, we found that vaccination of older adults would prevent hospitalizations by a median of 35–64% across the countries studied here. Vaccination of pregnant women could avert infant hospitalizations by 5–50%. Reductions in RSV-related mortality mirrored those estimated for hospitalizations. While substantial hospitalization costs could be averted, the impact of vaccination depends critically on uptake rates. Enhancing uptake and accessibility is crucial for maximizing the real-world impact of vaccination on reducing RSV burden among vulnerable populations.

## Abstract (web summary)

An individual-based mathematical model evaluating the public health benefits of RSVpreF vaccination showed that vaccinating older adults and pregnant women can substantially reduce respiratory syncytial virus (RSV)-related hospitalizations and deaths, averting substantial inpatient costs, with the impact varying across countries and influenced by both uptake rates and the local epidemiological characteristics of RSV seasons.

## Main

Respiratory syncytial virus (RSV) remains a leading cause worldwide of acute lower respiratory tract illness (LRTI) among children under 5 years of age^1,2^, with the highest burden occurring within the first 6 months of life^3^. Up to 2% of young children in high-income countries are hospitalized for the management of RSV-associated illnesses, including bronchiolitis, pneumonia, apnea and difficulty in feeding^4,5^. RSV can also cause serious illness in older adults, especially among those with comorbidities and risk factors^6^, often leading to severe outcomes and hospitalization. A meta-analysis of high-income countries reported a case-fatality rate of 7% in older adults admitted with RSV-associated LRTI^7^. The burden of RSV has been comparable to that of seasonal influenza among older adults^8^, resulting in substantial short- and long-term healthcare costs and productivity losses^9–12^.

Despite continuous efforts to develop RSV vaccines since the 1960s, no vaccine was sufficiently promising to enter phase 3 clinical trials until recently. Historically, RSV prevention among infants has relied on palivizumab, an expensive, short-term monoclonal antibody administered in five monthly doses to high-risk infants each RSV season^13^. Leveraging advances in structure-based vaccinology, the most immunogenic epitopes and conformations were identified to inform the design of vaccines and long-acting monoclonal antibodies^14^. Notably, a protein-based vaccine (Abrysvo) is now available to immunize pregnant women between 32 and 36 weeks of gestational age to protect infants through the first 90 days of life after birth^15^. In addition, Prefusion F protein-based (RSVpreF) vaccines (Abrysvo and Arexvy) have become available for the prevention of RSV disease in older adults^16,17^. Alongside these vaccines, a long-acting monoclonal antibody (nirsevimab) is approved for infant immunization against RSV-related LRTI in several countries^18,19^. Although nirsevimab provides direct, nonactive protection for infants, the lower costs of RSVpreF vaccination^20^ may provide a more affordable strategy for reducing disease burden in older adults, pregnant women and infants.

With the availability of RSVpreF vaccines, quantification of health benefits is essential to inform vaccination strategies^12,21^. In this study, we developed an individual-based simulation model of RSV transmission dynamics with vaccination to estimate the number of RSV-related hospitalizations and deaths averted among infants and adults aged 60 years or older in 13 high-income countries from different regions across the world, including the United States, Canada, United Kingdom, Germany, France, Italy, the Netherlands, Sweden, Spain, Ireland, Israel, Japan and Australia. We considered vaccine efficacy against severe outcomes (that is, preventing hospitalization), but assumed no vaccine-induced protection against infection or transmission. Incorporating country-specific demographics, age-dependent contact patterns and estimates of disease burden, we simulated the model with influenza-like vaccination uptake rates to provide a multinational perspective on the impact of RSVpreF vaccination programs in these countries. Using model estimates for each country, we further calculated the direct costs of RSV-related hospitalizations and the years of life lost (YLL) averted by vaccination.

## Results

**Extended Data Fig. 1:**
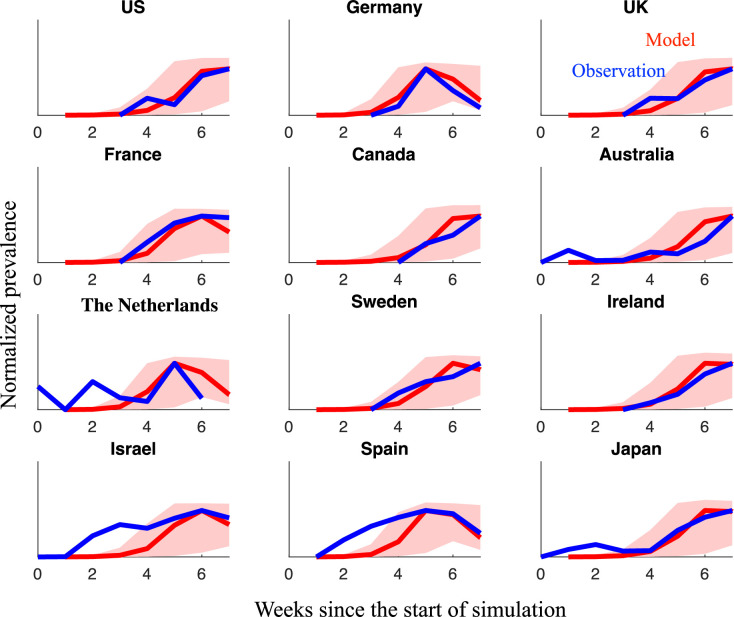
Performance of the model with estimated reproduction numbers compared with observed weekly positivity rates of RSV in countries studied. For each country, weekly RSV positivity rates were collected for the 2018–2019 season^56^, with the estimates of the effective reproduction number (Supplementary Table 6). We normalized both the positivity rates (blue curves) and the median (and 95% CrI) estimates of average weekly prevalence (red curves and shaded areas) based on 500 independent stochastic simulations in the model.

We estimated the effective reproduction number, *R*_e_ (that is, the average number of secondary infections generated by an RSV case), using weekly positivity rates reported for the 2018–2019 RSV seasons across the countries studied here. Australia showed the lowest mean *R*_e_ value at 1.29, while Germany exhibited the highest at 1.99 (Supplementary Table 6). Calibrating the transmission dynamic model to country-specific *R*_e_ values (Extended Data Fig. 1), we ran stochastic simulations to project the total number of hospitalizations and deaths in the absence of vaccination as the baseline scenario.

Without vaccination, the annual burden of RSV varied substantially across the 13 countries studied, with hospitalizations ranging from a median of 27.9 (95% uncertainty range (UR): 27.0–28.7) per 100,000 older adults in Israel to 214.0 (95% UR: 204.7–222.1) in the United States. Similarly, RSV-related mortality per 100,000 population of older adults varied from a median of 1.8 (95% UR: 1.7–1.8) in Israel to 20.8 (95% UR: 19.9–21.7) in Canada.

Among the infant population, Ireland had the lowest estimated hospitalization rate, with a median of 520.7 (95% UR: 0–813.5) per 100,000 infants, while the Netherlands exhibited the highest at 3,373.0 (95% UR: 3,104.4–3,611.8). RSV-related mortality also varied substantially, ranging from a median of 0.14 (95% UR: 0.12–0.16) per 100,000 infants in Australia to 3.6 (95% UR: 3.3–3.9) in Israel. Across the countries studied, estimated YLL exceeded 280.2 (95% UR: 268.8–290.6) per 100,000 older adults and 311.5 (95% UR: 271.8–377.9) per 100,000 infants.

### Health outcomes with vaccination

We applied the calibrated model specific to each country and implemented vaccination programs targeting older adults and pregnant women, mirroring the reported vaccine coverage of these population groups for seasonal influenza preceding the COVID-19 pandemic (Supplementary Tables 7 and 8). Among older adults, averted hospitalizations attributed to RSV vaccination ranged from a median of 15.0 (95% UR: 13.9–16.1) per 100,000 population in Israel to 135.0 (95% UR: 128.9– 142.4) in Canada (Table 1). The corresponding vaccination uptake rates were reported at 59.8% in Israel and 70.0% in Canada (Supplementary Table 8). The estimated numbers of RSV-related deaths averted by RSV vaccination varied, with the lowest at 1.0 (95% UR: 0.9–1.0) and the highest at 11.2 (95% UR: 10.5–11.8) per 100,000 population of older adults, in Israel and Canada, respectively. The reduction in deaths attributed to RSV vaccination varied from 35.2% in Germany to 64.5% in the United Kingdom (Table 1). Based on averted deaths among older adults in each country, we estimated YLL prevented by RSV vaccination, which ranged from 21.5 (95% UR: 19.7–23.2) per 100,000 population in Israel to 168.7 (95% UR: 158.6–177.9) in the United States (Table 1).

**Table 1:** Projected hospitalizations, deaths, YLL and direct costs of hospitalizations averted per 100,000 older adults attributed to RSV vaccination, with country-specific influenza-like vaccination coverage among adults aged 60 years and above

Country	Estimate (95% UR)				
	Hospitalization averted	Death averted	YLL averted	Costs averted (×$US1,000)	Percentage of death averted
United States	128.69 (121.15–135.69)	11.17 (10.52–11.78)	168.71 (158.63–177.88)	2,538 (2,385–2,676)	60.26 (57.54–62.61)
Germany	42.15 (39.91–44.57)	3.12 (2.95–3.33)	37.34 (35.16–39.36)	303 (285–320)	35.20 (33.55–37.11)
United Kingdom	98.31 (94.22–102.51)	7.24 (6.92–7.57)	91.64 (87.49–95.31)	81 (78–85)	64.49 (62.40–66.46)
France	56.39 (54.21–59.03)	3.72 (3.58–3.90)	57.48 (55.22–60.33)	202 (194–211)	46.68 (44.89–48.60)
Italy	55.18 (51.28–59.08)	4.18 (3.86–4.50)	54.54 (50.68–58.62)	349 (325–374)	47.62 (45.08–49.82)
Canada	135.03 (128.88–142.37)	13.03 (12.40–13.79)	161.91 (153.84–170.67)	1,584 (1,507–1678)	62.93 (60.65–65.23)
Australia	81.50 (74.69–87.65)	3.68 (3.37–3.97)	62.86 (57.77 – 67.85)	1,395 (1,279–1,501)	50.49 (47.67–53.32)
the Netherlands	48.00 (45.97–49.88)	3.43 (3.29–3.56)	49.26 (47.18–51.21)	164 (157–171)	48.68 (46.75–50.37)
Sweden	45.59 (43.02–48.32)	3.51 (3.30–3.72)	41.26 (38.85–43.84)	855 (807–906)	46.72 (44.49–49.12)
Ireland	66.86 (1.73–85.32)	4.71 (0.13–6.07)	67.02 (1.55–85.71)	263 (7–336)	60.53 (52.70–67.74)
Israel	15.01 (13.86–16.13)	0.96 (0.89–1.03)	21.48 (19.69–23.20)	45 (42 –49)	53.82 (50.12–57.15)
Spain	49.30 (44.17–54.86)	3.69 (3.26–4.11)	51.42 (46.33–57.18)	141 (127–157)	49.50 (46.10–53.22)
Japan	70.43 (66.67–74.38)	3.92 (3.71–4.14)	58.76 (55.81–62.14)	236 (223–249)	44.88 (43.10–46.77)

Baseline scenario is given in Supplementary Table 13.

Among pregnant women, the highest vaccination uptake rates were observed in Australia (61%) and Ireland (61.7%) (Supplementary Table 7). Compared with the no-vaccine scenario, vaccination of pregnant women would avert a median of 1,060.9 (95% UR: 599.0–1,335.3) RSV-related hospitalizations per 100,000 infants in Australia, representing the most substantial reduction per capita among all countries studied here (Table 2). With a 47.4% vaccination coverage of pregnant women (Supplementary Table 7), Japan had the largest number of RSV-related mortalities averted, with a median of 1.3 (95% UR: 0.6–1.8) per 100,000 infants (Table 2). The reduction in deaths attributed to RSV vaccination varied from 4.8% in France to 49.7% in Ireland (Table 2).

**Table 2:** Projected hospitalizations, deaths, YLL and direct costs of hospitalizations averted per 100,000 infants attributed to RSV vaccination, with country-specific influenza-like vaccination coverage among pregnant women

Country	Estimate (95% UR)				
	Hospitalization averted	Death averted	YLL averted	Costs averted (×$US1,000)	Percentage of death averted
United States	932.12 (476.13–1,230.60)	0.93 (0.48–1.23)	73.54 (37.57–97.09)	9,521 (4,863–12,569)	38.41 (20.65– 6.90)
Germany	283.88 (93.12–407.71)	0.26 (0.08–0.37)	20.44 (6.70–29.35)	1,310 (430–1,881)	8.93 (2.88–13.04)
United Kingdom	809.32 (412.97–1,066.44)	0.73 (0.37–0.96)	58.96 (30.09–77.70)	1,131 (577–1,490)	28.89 (14.73–36.18)
France	156.38 (31.21–241.55)	0.14 (0.03–0.22)	11.57 (2.31–17.87)	683 (136–1,056)	4.82 (0.96–7.39)
Italy	228.13 (56.28–394.03)	0.21 (0.05–0.35)	17.04 (4.20–29.42)	1,444 (356–2,494)	10.82 (3.16–18.07)
Canada	698.84 (368.01–999.84)	0.70 (0.37–1.00)	57.28 (30.17–81.96)	5,922 (3,118–8,472)	32.08 (17.87–43.04)
Australia	1,060.90 (599.02–1,335.29)	0.06 (0.04–0.08)	5.30 (2.99–6.67)	18,163 (10,255–22,860)	45.44 (25.91–52.54)
the Netherlands	587.33 (241.23–784.54)	0.53 (0.22–0.71)	43.24 (17.76–57.75)	2,009 (825–2,684)	17.81 (7.39–23.51)
Sweden	583.23 (243.60–774.10)	0.52 (0.22–0.70)	43.25 (18.07–57.41)	10,939 (4,569–14,518)	20.83 (8.91–26.79)
Ireland	248.86 (0–437.25)	0.22 (0–0.39)	18.33 (0–32.21)	980 (0–1,721)	49.72 (28.88–70.46)
Israel	655.78 (325.50–826.48)	0.79 (0.39–0.99)	65.02 (32.27–81.94)	1,981 (983–2,497)	21.95 (10.92–26.93)
Spain	357.60 (159.77–589.54)	0.32 (0.14–0.53)	26.78 (11.97–44.15)	964 (431–1,589)	30.51 (15.22–48.10)
Japan	966.42 (461.99–1,261.15)	1.35 (0.65–1.77)	114.58 (54.78–149.53)	3,232 (1,545–4,217)	35.55 (16.80–43.89)

Baseline scenario is given in Supplementary Table 13.

**Fig. 1:**
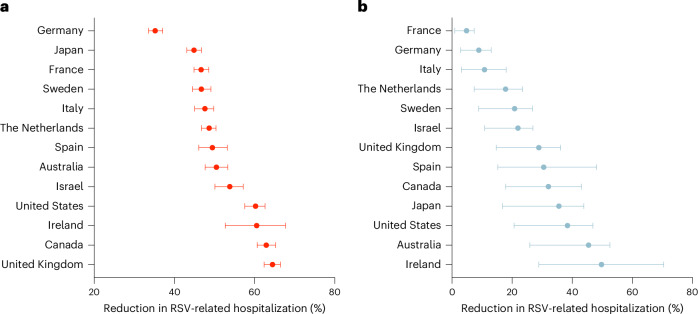
Estimated reduction in RSV-related hospitalizations among older adults and infants attributable to RSV vaccination. **a**,**b**, Estimated reduction in older adults (**a**) and infants (**b**). Error bars represent median values and 95% UR of 500 stochastic simulations with vaccination of older adults (**a**) and pregnant women (**b**).

Simulating vaccination scenarios, we calculated the relative reduction in hospitalizations for each country (Fig. 1). The reduction in hospitalization achieved by vaccination of older adults ranged from 35.2% (95% UR: 33.5–37.1%) in Germany to 64.5% (95% UR: 62.4–66.5%) in the United Kingdom (Fig. 1a). In the case of vaccination of pregnant women, France showed the lowest reduction in RSV-related hospitalizations among infants, with a reduction of 4.8% (95% UR: 1.0–7.4%), while Ireland exhibited the highest reduction, of 49.7% (95% UR: 28.9–70.5%) (Fig. 1b), reflecting vaccination coverage of pregnant women (Supplementary Table 7).

### Hospitalization costs averted by vaccination

**Fig. 2:**
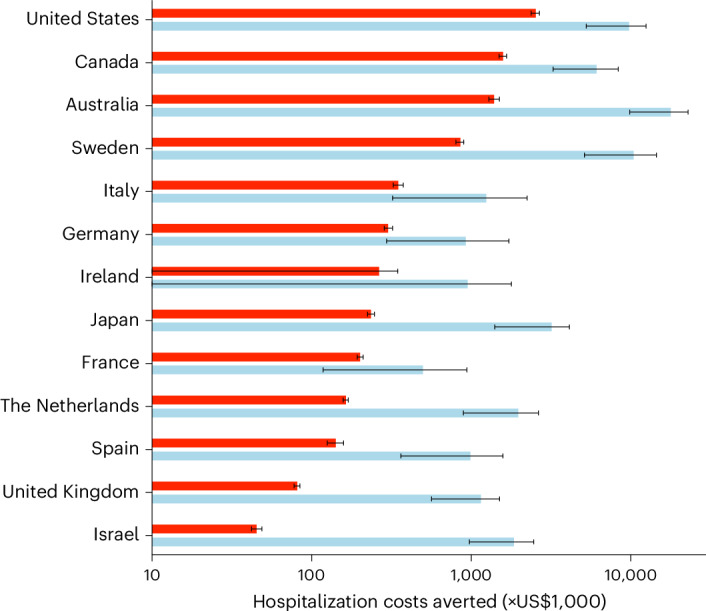
Estimated direct costs of RSV-related hospitalizations averted by vaccination. Assuming uptake rates similar to seasonal influenza vaccination in each country (Supplementary Tables 7 and 8), the vaccination scenario is compared with the baseline scenario (without vaccination), with country-specific reproduction numbers estimated for the 2018–2019 RSV season (Supplementary Table 6). Colored bars represent median values of estimates for vaccination of older adults (red, per 100,000 older adults) and pregnant women (cyan, per 100,000 infants), with error bars indicating 95% UR from 500 stochastic simulations.

Using country-specific costs of RSV-related hospitalization, we calculated the direct costs averted by RSV vaccination (Methods). Costs averted by vaccination of older adults ranged from US$45,202 (95% UR: US$42,100–48,803) in Israel to US$2,540,408 (95% UR: US$2,375,525–2,679,474) in the United States per 100,000 population of adults aged 60 years or older (Fig. 2, red bars). Hospitalization costs averted per 100,000 infants attributed to the vaccination of pregnant women were lowest in France, at US$498,163 (95% UR: US$117,922–940,689) and highest in Australia, at US$17,855,740 (95% UR: US$9,868,897–22,865,179) (Fig. 2, cyan bars).

### Secondary analyses

**Extended Data Fig. 2:**
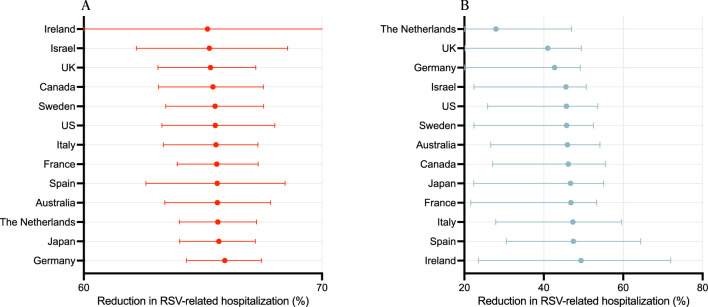
Estimated reduction of RSV-related hospitalizations among older adults (A) and infants (B) attributable to RSV vaccination in Scenario V1. Error bars represent median values and 95% uncertainty ranges of the 500 stochastic simulations with vaccination of older adults (A, red) and pregnant women (B, cyan).

**Extended Data Fig. 3:**
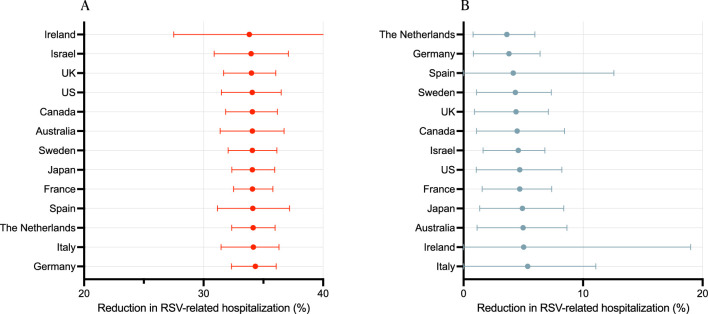
Estimated reduction of RSV-related hospitalizations among older adults (A) and infants (B) attributable to RSV vaccination in Scenario V2. Error bars represent median values and 95% uncertainty ranges of the 500 stochastic simulations with vaccination of older adults (A, red) and pregnant women (B, cyan).

To investigate the effect of vaccination coverage on reduction of RSV burden and associated hospitalization costs, we conducted additional scenario analyses by varying uptake rates in each country (Supplementary Tables 14–19). Setting the vaccination coverage to 73% for older adults and 62% for pregnant women in all countries (V1 scenario in Supplementary Table 13), the reduction in hospitalizations (compared with baseline) was similar across the countries studied, with overlapping uncertainty ranges of 62–73% for older adults and 18–72% for infants (Extended Data Fig. 2). For the lowest coverages of 38% for older adults and 7% for pregnant women (V2 scenario in Supplementary Table 13), the reduction in hospitalizations was substantially lower but still consistent across countries, with overlapping uncertainty ranges of 28–41% for older adults and 0–19% for infants (Extended Data Fig. 3).

**Extended Data Fig. 4:**
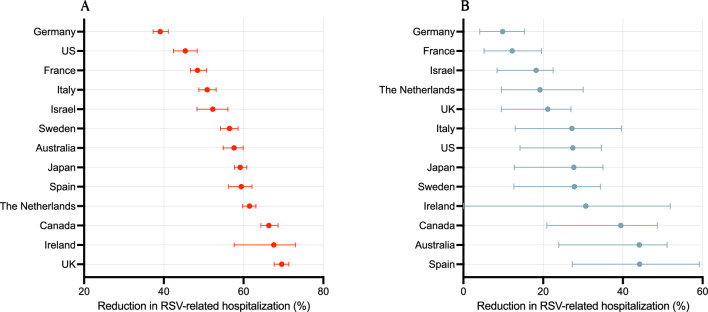
Estimated reduction of RSV-related hospitalizations among older adults (A) and infants (B) attributable to RSV vaccination in Scenario V3. Error bars represent median values and 95% uncertainty ranges of the 500 stochastic simulations with vaccination of older adults (A, red) and pregnant women (B, cyan).

We further considered country-specific vaccination coverage of seasonal influenza (V3 scenario in Supplementary Table 13) reported during and after the COVID-19 pandemic (Supplementary Tables 7 and 8). Simulating the model with the vaccination of older adults, we found trends in the reduction of hospitalizations similar to those derived in the baseline scenario with country-specific vaccination coverage before the COVID-19 pandemic. The median reduction in hospitalizations ranged from 39% in Germany to 70% in the United Kingdom (Extended Data Fig. 4). In regard to vaccination of pregnant women, median reduction in hospitalizations among infants varied from 10% in Germany to 44% in Spain (Extended Data Fig. 4).

## Discussion

The availability of RSVpreF vaccines provides an important public health measure to mitigate the burden of RSV disease among infants and older adults. Our analysis indicates that vaccination can substantially reduce RSV-related hospitalizations and deaths, averting inpatient costs. However, this impact varies across countries and is influenced by both uptake rates and local epidemiological characteristics of RSV seasons. For example, Germany has one of the highest burdens of RSV among European countries, with an annual average of 127 hospitalizations per 100,000 adults aged 65 years or older^22^, incurring substantial direct and indirect healthcare costs^23^. Given the low influenza vaccination coverage of 38.8% among older adults in Germany, the estimated reduction in RSV-related hospitalizations remains under 40% in this population. Similarly, in countries such as France, Germany and Italy, where maternal immunization uptake rates are low, the estimated reductions in infant hospitalizations remain under 20% (Fig. 1).

Our secondary analyses show that the outcomes of RSV vaccination may vary across populations, even under the assumption of similar uptake rates and transmissibility; for example, the United States, Canada, Ireland and Japan have similar reproduction numbers, between 1.4 and 1.5. When the vaccination coverage was set to 73% for older adults and 62% for pregnant women across these countries, the projected hospitalizations averted per 100,000 population of older adults varied from 72.5 (95% UR: 1.7–93.0) in Ireland to 140.8 (95% UR: 133.7–148.7) in Canada, indicating the impact of demographic characteristics on vaccination outcomes. However, the relative reductions in outcomes were similar, despite the varying burden of RSV among these countries.

Our model, calibrated to country-specific reproduction numbers, produces hospitalization rates consistent with those published in the literature. For instance, one systematic analysis of global, regional and national disease burden reported an overall annual rate of 17.8 (95% UR: 11.9–26.6) hospitalizations per 1,000 infants in high-income countries (using studies with quality scores >0.6)^2^. In the absence of vaccination, hospitalization rates estimated in our model per 1,000 infants range from 5.2 (95% UR: 0–8.1) in Ireland to 33.7 (95% UR: 31.0–36.1) in the Netherlands. Similarly, the model generates hospitalization rates per 100,000 older adults ranging from 97.7 (95% UR: 94.5–100.6) in Sweden to 214.0 (95% UR: 204.7–222.1) in the United States, largely consistent with the unadjusted annual hospitalization rate of 157 (95% CI: 98–252) in industrialized countries^7^.

In alignment with previous studies^24–27^, our analysis demonstrates the potential for substantial health benefits from RSVpreF vaccines, assuming high vaccination uptake rates. However, uptake rates will depend on various factors such as vaccine acceptability, out-of-pocket costs for receiving RSV vaccines, the inclusion of eligible population groups in publicly funded programs and specific recommendations for program implementation in different countries, which are influenced by cost-effectiveness evaluations of these vaccines^12,21,28–30^. Recommendations are likely to consider other RSV interventions, such as nirsevimab, which offers an alternative to maternal vaccination by providing passive immunity to infants. For instance, a recent cost-effectiveness analysis of RSV preventive measures in Canada found that a combined strategy of vaccinating pregnant women with RSVpreF vaccines and immunizing high-risk infants with nirsevimab during the RSV season was cost effective, while being comparable to an all-infant, nirsevimab-only program in reducing infant hospitalizations and mortality^21^. However, despite regulatory approvals for the use of nirsevimab in several countries studied here, program implementation for infant immunization remains challenging due to limited supply and high costs, even in high-income countries^31–36^. Recommendations may also vary across countries, influencing the uptake and prioritization of RSVpreF vaccines and nirsevimab. For example, in the United States, infants whose mothers have been immunized with RSVpreF vaccines may not receive nirsevimab (depending on risk factors and gestational age)^37^, making nirsevimab uptake uncertain and dependent on RSVpreF vaccine uptake in pregnant women. Given these uncertainties and varying recommendations, and considering additional factors including costs, our analysis did not include nirsevimab for infant immunization.

Our study has several limitations. First, we implemented uptake rates at the beginning of model simulations, corresponding to the start of the RSV season in each country. However, vaccination probably occurs throughout the season, and effective uptake rates at the onset of the RSV season may differ from those used in our analysis. In addition, we also used reported vaccine efficacy estimates from clinical trials. However, the real-world effectiveness of these vaccines remains unknown and would be influenced by the characteristics of eligible population, comorbidities and other RSV-associated risk factors. Second, we focused only on the direct benefits of vaccination against severe disease outcomes (hospitalization and death), and on the reduction of costs associated with RSV-related hospitalizations. Additional benefits of vaccination, such as reduced rates of medically attended symptomatic RSV for outpatient care, were not considered in this study^12,21,28,38,39^. Furthermore, while current evidence is limited, vaccination may reduce transmission and circulation of the virus due to potential herd immunity effects. Without accounting for these factors, our results regarding the health benefits of vaccination are probably conservative. However, in the absence of data on the indirect effects of vaccination against infection or transmission, our model assumptions relied on existing evidence that RSV infection does not confer durable immunity and that reinfection is common^40^. Third, we evaluated RSVpreF vaccination over a 1-year time horizon consisting of a single RSV season. However, these vaccines have shown protective efficacy against severe RSV LRTI over two seasons^41–43^, which could affect uptake rates. Fourth, our estimates of reproduction numbers rely on reported RSV positivity rates in the countries studied. However, various factors can influence such rates, including risks and care-seeking behavior, testing practices and seasonal forcing. Fifth, our analysis uses hospitalization costs from published estimates, which may be influenced by the population study as well as the utilization of critical care resources by inpatients. Sixth, we did not account for the costs of vaccination programs in our analysis, and thus net direct healthcare cost savings would differ from those estimated here. Costs of vaccination programs would include vaccine transportation, storage, administration, promotion and outreach, wastage and the price per dose of vaccines, which varies by country due to manufacturers’ negotiations. Finally, the cost effectiveness of RSVpreF vaccines and the impact of nirsevimab alongside vaccination were not evaluated in our analysis. Such evaluations would require an extension of the model structure to include country-specific inputs, such as the outpatient burden of RSV and associated costs, recommendations for use of these interventions (for example, risk factors and gestational age of infants) and estimates of productivity losses due to RSV-related illness^12,21,26,28,44^.

Given the recent introduction of RSV vaccines in 2023, there is limited real-world experience with vaccine program implementation. Although we used country-specific influenza vaccination coverage in our baseline analysis, recent estimates indicate a substantially lower uptake of RSVpreF vaccines during the 2023–2024 season^45–48^. Addressing vaccine hesitancy, awareness and accessibility should improve uptake rates, thereby enhancing the impact of RSV vaccination campaigns.

Within a few years, the landscape for the prevention of RSV-associated illness has significantly changed, and practical strategies to protect infants and older adults have become available. Our analysis underscores the importance of these advancements, demonstrating that the use of RSVpreF vaccines could substantially reduce the burden of RSV disease. As new data and evidence on the real-world effectiveness of these vaccines accumulate, additional studies can inform optimal vaccination scenarios in different populations.

## Methods

### Ethics

Our study utilized publicly available data sources and published estimates and thus required no ethics approval.

### Model structure

We developed an individual-based model of RSV transmission dynamics, linking disease spread at the population level with temporal changes in viral load at the individual level. We parameterized the model with demographics of each country (Supplementary Tables 1–3) and calibrated it to the country-specific effective reproduction number estimated using epidemiological data from the 2018–2019 RSV season. We considered vaccination coverage of older adults and pregnant women similar to those reported for seasonal influenza in each country, and simulated additional scenarios with varying vaccination coverage to estimate the impact of RSV vaccines on reducing disease burden in terms of hospitalizations and deaths averted.

### Individual-based network

**Extended Data Fig. 5:**
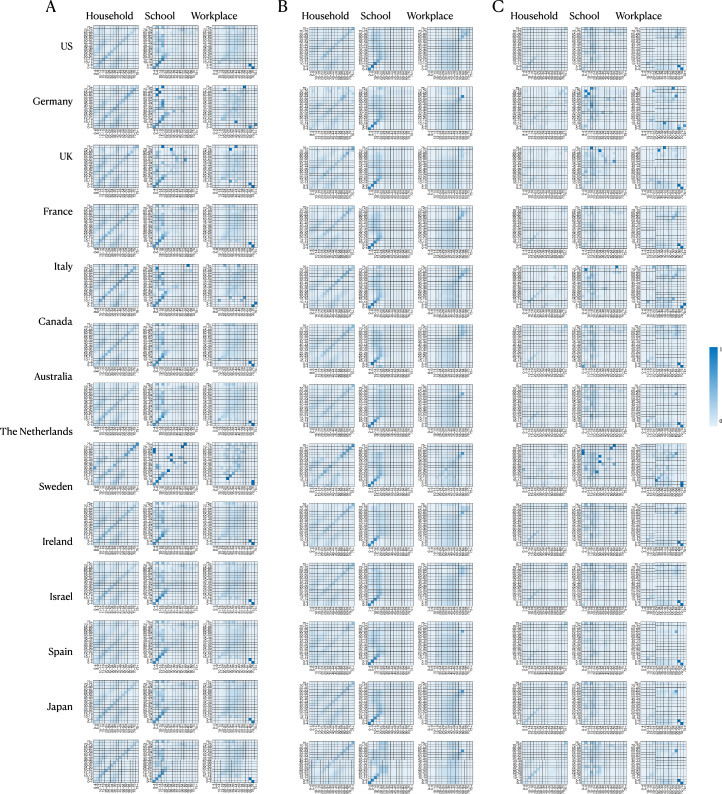
Age-specific contact patterns. Contact patterns generated for 5-year age groups across settings of households, schools, and workplaces using (**A**) survey ^60^, (**B**) the median values of 500 simulated demographics from the model, and (**C**) the difference between (**A**) and (**B**). The color in each cell denotes the proportion of daily contacts for individuals in an age group (horizontal) with another age group (vertical). Similarity of contact matrices was evaluated using the Frobenius norm, reported in Supplementary Table 3.

**Extended Data Fig. 6:**
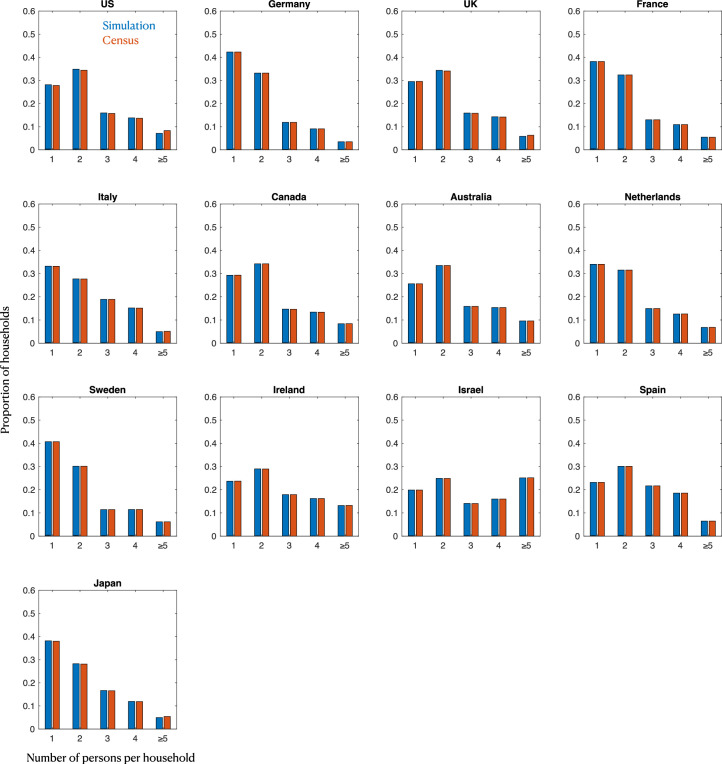
Household size distribution. We compared the distribution of household sizes from census data (Supplementary Table 2, red bar) with those of simulated demographics for each country. Each individual in the household is completely interconnected with one another. The median value of 500 simulated demographics from the model are shown with blue bars.

The model simulates the spread of disease through an individual-based network. We considered the demographics and household structure of each country, scaled down to 10,000 individuals in the model. Following previous work^49,50^, we constructed realistic, synthetic contact networks of individuals (Extended Data Fig. 5) employing the open-source, data-driven model of SynthPops, using households, workplaces, schools and other public settings and satisfying population demographics (that is, age distribution and household size (Extended Data Fig. 6)) informed by census and survey data for the countries studied (Supplementary Table 2). Briefly, SynthPops leverages census data and survey information to define characteristics including age, household size, school enrollment and employment rates. Age-specific contact matrices are utilized to model individuals and their expected interactions with others within a multilayered network structure. Within each social layer, individuals are assigned daily contact numbers with others across age groups, drawn from a Poisson distribution whose mean is determined by age-specific contact matrices. For instance, the SynthPops algorithm assigns household size according to a prespecified distribution, and then selects an age for the reference individual in each household based on household size and age distribution. Other household members and their ages are determined from demographic distributions based on age-specific contact matrices relative to the age of the reference individual within the household^49,50^. We validated our model in reproducing the demographics of the countries studied here by comparison of the simulated contact patterns with census and survey data in population settings of households, schools and workplaces (Extended Data Fig. 5). The similarity of contact matrices was evaluated using the Frobenius norm, averaging >500 independent realizations (Supplementary Table 3). We also compared simulated and data-driven distribution of household sizes for each country (Extended Data Fig. 6).

### Epidemiological states

**Extended Data Fig. 7:**
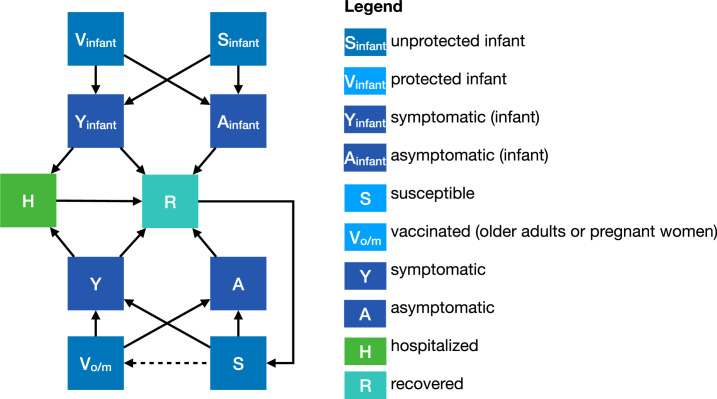
Model description. Schematic diagram of the model for transmission dynamics of RSV and vaccination.

Individuals in the model were classified as either infants under 1 year of age, pregnant women (who are eligible for RSV maternal immunization), adults aged 60 years or older (who are eligible to receive the RSV vaccine) or other individuals (Extended Data Fig. 7). We considered age-specific rates of developing symptomatic RSV disease following infection (Supplementary Table 4). Conservatively, we assumed that RSV vaccination did not affect susceptibility to infection, but reduced the risk of severe disease and hospitalization^21,39,43,51,52^. Assuming that maternal protection conferred by vaccination of pregnant women did not affect rates of RSV infection, we classified infected infants as either asymptomatic or symptomatic and who may be hospitalized based on estimated rates for each country (Supplementary Table 5), while accounting for the efficacy of maternal protection against RSV LRTI. Infection among other individuals in the model was also classified as either asymptomatic or symptomatic with the associated hospitalization rates (Supplementary Tables 4 and 5).

### Transmission dynamics

**Extended Data Fig. 8:**
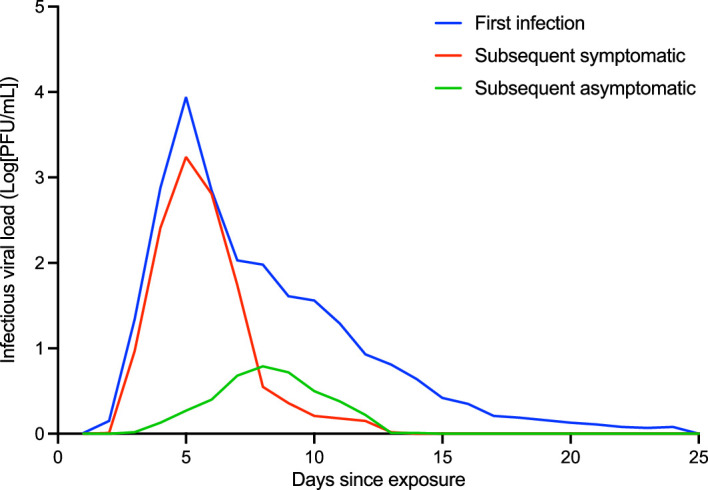
Viral load dynamics. Viral load (log[PRU/mL]) during the primary and subsequent RSV infections^54^.

We simulated the model by introducing an infected individual in the state of symptomatic infection, linking the transmission dynamics between individuals to temporal changes in viral load in infected individuals. The logarithm of viral load has been shown to correlate with the transmissibility of RSV^53,54^. Specifically, the transmission rate, denoted by ${\beta }_{j}$, varies over time for individual *j* who is infected at time *T*. At any time *t*, ${\beta }_{j}$ can be estimated by

$${\beta }_{j}(t)=\phi \log ({V}_{j}(t-T\,)),$$

where $\phi$ is the adjusting factor for baseline transmission rate, estimated from the effective reproduction number, and *V*_*j*_(*t* − *T*) is viral load (Extended Data Fig. 8) during primary or subsequent infections of individual *j* at time *t* ≥ *T* (ref. ^54^). We considered this functional form to account for temporal changes in RSV transmission due to differences in viral load during symptomatic and asymptomatic states of infection^54^. Susceptible individuals become infected (and move to the asymptomatic or symptomatic state) at rate *λ*, given by

$$\lambda =\sum _{j}{\beta }_{j}(t){{/}}N,$$

where *N* is the total number of individuals and ${\beta }_{j}(t)$ is the transmission rate of the *j*th infectious individual at time *t*. We assumed that ${\beta }_{j}(t)$ is the product of a population-wide scaling factor $\phi$ matched to the effective reproduction number and viral load of the infected individual at time *t* when the contact occurs. Conservatively, we assumed no reduction of transmission for individuals who were infected despite vaccination. Hospitalized cases were assumed to be isolated and did not contribute to infection transmission.

Initializing the model, we assumed that the infant population (*S*_infant_) is immunologically naive to RSV infection, while other individuals are susceptible (*S*) but have previously experienced RSV infection. The risk of infection and disease may differ among Individuals due to their social activities and age-specific contact patterns in different settings (Extended Data Fig. 5). The susceptibility of individuals older than 1 year of age was assumed to be 0.89 (95% credible interval (CrI): 0.85–0.93) for a secondary infection compared with a primary infection in immunologically naive infants, 0.81 (95% CrI: 0.74–0.85) for a tertiary infection compared to a secondary infection and 0.33 (95% CrI: 0.31–0.37) for a subsequent infection compared with tertiary infection^26,55^. The susceptibility level was sampled for each individual independently from the corresponding beta-distributions (Supplementary Table 4)^26,55^. We assumed that, except for factors that affect the risk of infection (that is, susceptibility of individuals or viral load of the infected individuals at the time of contact), transmission dynamics are independent of the social setting within which contacts occur.

### Effective reproduction number

For each country, we collected weekly positivity rates of RSV for the 2018–2019 season and detected season periods using the moving epidemic method (Extended Data Fig. 1)^56^. We then estimated exponential growth rate, ${\varLambda }_{w}$, between the second week following the start of the season with weekly increments up the week before the peak of the RSV season. For each additional week from the onset of the RSV season, the effective reproduction number (*R*_e_) was calculated by ${R}_{{\mathrm{e}}}=1+({\root{7}\of{{\varLambda }_{w}+1}}-1)D$ (ref. ^57^), where *D* is the average infectious period, set to 7.72 days^58^. For calculation of *R*_e_ in a single simulation, we derived the average number of secondary cases produced by one initial infection introduced at the start of the simulation^59^. We computed mean *R*_e_ over 500 independent stochastic outbreak simulations (Supplementary Table 6). Model performance in simulating RSV prevalence was consistent with the observed trends in RSV positivity rates in each country (Extended Data Fig. 1).

### Vaccination scenarios

In the main analysis, vaccination uptake rates for pregnant women and older adults against RSV were set to those reported for 2018–2019 seasonal influenza in the countries studied here (Supplementary Tables 7 and 8). We performed additional analyses by varying vaccination coverage among these population groups with estimates available for influenza vaccination coverage during and after the COVID-19 pandemic (Supplementary Table 8). We considered two scenarios: vaccination of pregnant women (S1) and older adults (S2). These scenarios were compared with the baseline scenario in the absence of vaccination, reflecting the 2018–2019 RSV season. Because vaccination was assumed (conservatively) to provide no protection against RSV infection, vaccination of both older adults and pregnant women results in cumulative outcomes as estimated in scenarios S1 and S2.

### Vaccine efficacy

The efficacy of Abrysvo against severe LRTI was used to parameterize the model for protection of vaccinated individuals against hospitalization^39,43,51,52^. We assumed no vaccine-induced protection against infection or transmission, and thus transmission dynamics were not affected by vaccination. For infants whose mothers were vaccinated during the last trimester before birth, the risk of hospitalization was reduced by an average of 67.7% for the first 90 days of life compared with infants lacking maternal immunization^21,39^. This protection efficacy further decreased to an average of 33.9% starting at month 4 after birth and continuing to 10 months of age^21^. For older adults and pregnant women who were vaccinated with Abrysvo, the protection efficacy against severe LRTI (hospitalization) is estimated at an average of 88.9% throughout the first RSV season postvaccination^43,51^. Using these mean values of vaccine efficacy and the corresponding 95% confidence intervals, we derived beta-distributions by minimizing mean squared errors. Vaccine efficacy was then sampled individually for infants, older adults and pregnant women from the associated distributions (Supplementary Table 9).

### Estimating outcomes

To estimate the health benefits of RSV vaccination, we conducted pairwise experiments in which stochastic simulations were repeatedly run for baseline and vaccination scenarios for a 1-year time horizon from the start of the season in each country. For each pair of simulations, we recorded the difference in total hospitalizations and deaths between baseline and vaccination scenarios and estimated the impact of vaccination in reducing these outcomes and averting YLL, based on the remaining life expectancy at the age of death due to RSV infection (Supplementary Table 10).

For a given vaccination scenario ($\tau$, corresponding to S1 or S2), the total numbers of hospitalizations (${H}_{\tau ,\alpha }$) and deaths (${D}_{\tau ,\alpha }$) at age *a* were calculated in each simulation using

$${H}_{\tau ,\alpha }={p}_{{{\mathrm{infect}}},{{\mathrm{hosp}}}}^{a}{I}_{\tau ,a}$$

$${D}_{\tau ,\alpha }={p}_{{{\mathrm{hosp}}},{{\mathrm{death}}}}^{a}{H}_{\tau ,a},$$

where ${p}_{{{\mathrm{infect}}},{{\mathrm{hosp}}}}^{a}$ is the proportion of total infected individuals (*I*_*a*_) of age *a* who were hospitalized, and ${p}_{{{\mathrm{hosp}}},{{\mathrm{death}}}}^{a}$ is the proportion of hospitalized individuals of age *a* who died (Supplementary Table 11).

### Estimating direct cost of hospitalization averted

We estimated the costs of RSV-related hospitalizations averted in vaccination scenario $\tau$ using

$${C}_{\tau }=\sum _{a}{c}_{H,\alpha }\left({H}_{\tau ,\alpha }-{H}_{0,\alpha }\right),$$

where ${c}_{H,\alpha }$ is the average cost of hospitalization per patient in age group *a* (Supplementary Table 12), and ${H}_{0,\alpha }$ represents the total number of hospitalized cases of age *a* in the baseline scenario without vaccination. All costs were converted and inflated to 2023 US dollars.

### Statistical analyses

We used the outcomes of 500 stochastic simulations to estimate median and 95% UR for reductions in RSV-related hospitalizations, deaths, YLL and direct healthcare costs attributed to vaccination in each country. All analyses were conducted using MATLAB 2023a.

### Reporting summary

Further information on research design is available in the Nature Portfolio Reporting Summary linked to this article.

## Online content

Any methods, additional references, Nature Portfolio reporting summaries, source data, extended data, supplementary information, acknowledgements, peer review information; details of author contributions and competing interests; and statements of data and code availability are available at 10.1038/s41591-024-03431-7.

## Supplementary information

Supplementary Information Supplementary Files 1 and 2.

Reporting Summary

## Data Availability

All data used in this study were derived from publicly available sources.

## Code availability

All codes used in this study can be accessed through GitHub (https://github.com/ZhanweiDU/RSV_Github).

## References

[1] Hall, C. B. et al. The burden of respiratory syncytial virus infection in young children. *N. Engl. J. Med.* **360**, 588–598 (2009). 19196675 10.1056/NEJMoa0804877 PMC4829966

[2] Li, Y. et al. Global, regional, and national disease burden estimates of acute lower respiratory infections due to respiratory syncytial virus in children younger than 5 years in 2019: a systematic analysis. *Lancet* **399**, 2047–2064 (2022). 35598608 10.1016/S0140-6736(22)00478-0 PMC7613574

[3] Glezen, W. P., Taber, L. H., Frank, A. L. & Kasel, J. A. Risk of primary infection and reinfection with respiratory syncytial virus. *Am. J. Dis. Child.* **140**, 543–546 (1986). 3706232 10.1001/archpedi.1986.02140200053026

[4] Shi, T. et al. Global, regional, and national disease burden estimates of acute lower respiratory infections due to respiratory syncytial virus in young children in 2015: a systematic review and modelling study. *Lancet* **390**, 946–958 (2017). 28689664 10.1016/S0140-6736(17)30938-8 PMC5592248

[5] Goldstein, E. et al. Hospitalizations associated with respiratory syncytial virus and influenza in children, including children diagnosed with asthma. *Epidemiology* **30**, 918–926 (2019). 31469696 10.1097/EDE.0000000000001092 PMC6768705

[6] Shi, T. et al. Disease burden estimates of respiratory syncytial virus related acute respiratory infections in adults with comorbidity: a systematic review and meta-analysis. *J. Infect. Dis.* **226**, S17–S21 (2022). 34522961 10.1093/infdis/jiab040

[7] Savic, M., Penders, Y., Shi, T., Branche, A. & Pirçon, J.-Y. Respiratory syncytial virus disease burden in adults aged 60 years and older in high-income countries: a systematic literature review and meta-analysis. *Influenza Other Respir. Viruses* **17**, e13031 (2023). 36369772 10.1111/irv.13031 PMC9835463

[8] Falsey, A. R., Hennessey, P. A., Formica, M. A., Cox, C. & Walsh, E. E. Respiratory syncytial virus infection in elderly and high-risk adults. *N. Engl. J. Med.* **352**, 1749–1759 (2005). 15858184 10.1056/NEJMoa043951

[9] Verelst, F., La, E. M., Graham, J. & Molnar, D. Leveraging time-use data to estimate market and non-market productivity losses due to respiratory syncytial virus (RSV) disease among adults aged ≥60 years in the United States (US). *Value Health* **26**, S120 (2023).

[10] Grace, M., Colosia, A., Wolowacz, S., Panozzo, C. & Ghaswalla, P. Economic burden of respiratory syncytial virus infection in adults: a systematic literature review. *J. Med. Econ.* **26**, 742–759 (2023). 37167068 10.1080/13696998.2023.2213125

[11] Mao, Z. et al. Economic burden and health-related quality of life of respiratory syncytial virus and influenza infection in European Community-dwelling older adults. *J. Infect. Dis.* **226**, S87–S94 (2022). 35961055 10.1093/infdis/jiac069

[12] Moghadas, S. M. et al. Cost-effectiveness of Prefusion F protein-based vaccines against respiratory syncytial virus disease for older adults in the United States. *Clin. Infect. Dis*. **78**, 1328–1335 (2024). 10.1093/cid/ciad658 PMC11093660 38035791

[13] Gutfraind, A., Galvani, A. P. & Meyers, L. A. Efficacy and optimization of palivizumab injection regimens against respiratory syncytial virus infection. *JAMA Pediatr.* **169**, 341–348 (2015). 25706618 10.1001/jamapediatrics.2014.3804 PMC4391881

[14] Graham, B. S. The journey to RSV vaccines - heralding an era of structure-based design. *N. Engl. J. Med.* **388**, 579–581 (2023). 36791157 10.1056/NEJMp2216358

[15] Office of the Commissioner. FDA approves first vaccine for pregnant individuals to prevent RSV in infants. US Food and Drug Administration www.fda.gov/news-events/press-announcements/fda-approves-first-vaccine-pregnant-individuals-prevent-rsv-infants (2023).

[16] Pfizer. Pfizer announces positive top-line data of phase 3 global maternal immunization trial for its bivalent respiratory syncytial virus (RSV) vaccine candidate. www.pfizer.com/news/press-release/press-release-detail/pfizer-announces-positive-top-line-data-phase-3-global

[17] Office of the Commissioner. FDA approves first Respiratory Syncytial Virus (RSV) vaccine. US Food and Drug Administration www.fda.gov/news-events/press-announcements/fda-approves-first-respiratory-syncytial-virus-rsv-vaccine (2022)

[18] AstraZeneca. Beyfortus approved in Japan for the prevention of RSV disease in infants. www.astrazeneca.com/media-centre/press-releases/2024/beyfortus-approved-in-japan-for-the-prevention-of-rsv-disease-in-infants.html (2024).

[19] Sanofi. FDA approves Beyfortus^TM^ (nirsevimab-alip) to protect infants against RSV disease. www.sanofi.com/en/media-room/press-releases/2023/2023-07-17-17-00-00-2705911 (2023)

[20] Centers for Disease Control and Prevention. CDC Current CDC vaccine price list www.cdc.gov/vaccines/programs/vfc/awardees/vaccine-management/price-list/index.html (2024).

[21] Shoukat, A. et al. Cost-effectiveness analysis of nirsevimab and maternal RSVpreF vaccine strategies for prevention of Respiratory Syncytial Virus disease among infants in Canada: a simulation study. *Lancet Reg. Health Am.* **28**, 100629 (2023). 38026446 10.1016/j.lana.2023.100629 PMC10663690

[22] Osei-Yeboah, R. et al. Estimation of the number of respiratory syncytial virus-associated hospitalizations in adults in the European Union. *J. Infect. Dis.* **228**, 1539–1548 (2023). 37246742 10.1093/infdis/jiad189 PMC10681866

[23] Niekler, P., Goettler, D., Liese, J. G. & Streng, A. Hospitalizations due to respiratory syncytial virus (RSV) infections in Germany: a nationwide clinical and direct cost data analysis (2010-2019). *Infection* **52**, 1715–1724 (2024). 37973718 10.1007/s15010-023-02122-8 PMC11499329

[24] Álvarez Aldean, J. et al. Cost-effectiveness analysis of maternal immunization with RSVpreF vaccine for the prevention of respiratory syncytial virus among infants in Spain. *Infect. Dis. Ther.* **13**, 1315–1331 (2024). 38733493 10.1007/s40121-024-00975-6 PMC11128416

[25] Postma, M. J. et al. Predicted public health and economic impact of respiratory syncytial virus vaccination with variable duration of protection for adults ≥60 years in Belgium. *Vaccines (Basel)* **11**, 990 (2023). 37243094 10.3390/vaccines11050990 PMC10223592

[26] Hodgson, D. et al. Protecting infants against RSV disease: an impact and cost-effectiveness comparison of long-acting monoclonal antibodies and maternal vaccination. *Lancet Reg. Health Eur.* **38**, 100829 (2024). 38476752 10.1016/j.lanepe.2023.100829 PMC10928299

[27] Molnar, D. et al. Public health impact of the adjuvanted RSVPreF3 vaccine for respiratory syncytial virus prevention among older adults in the United States. *Infect. Dis. Ther.* **13**, 827–844 (2024). 38507143 10.1007/s40121-024-00939-w PMC11058166

[28] Shoukat, A. et al. Impact and cost-effectiveness analyses of vaccination for prevention of respiratory syncytial virus disease among older adults in Ontario: a Canadian Immunization Research Network (CIRN) study. *Vaccine* **42**, 1768–1776 (2024). 38368226 10.1016/j.vaccine.2024.02.041

[29] Getaneh, A. M. et al. Cost-effectiveness of monoclonal antibody and maternal immunization against respiratory syncytial virus (RSV) in infants: evaluation for six European countries. *Vaccine* **41**, 1623–1631 (2023). 36737318 10.1016/j.vaccine.2023.01.058

[30] Baral, R., Higgins, D., Regan, K. & Pecenka, C. Impact and cost-effectiveness of potential interventions against infant respiratory syncytial virus (RSV) in 131 low-income and middle-income countries using a static cohort model. *BMJ Open* **11**, e046563 (2021). 33895717 10.1136/bmjopen-2020-046563 PMC8074564

[31] Centers for Disease Control and Prevention. Limited Availability of Nirsevimab in the United States—Interim CDC recommendations to protect infants from Respiratory Syncytial Virus (RSV) during the 2023–2024 Respiratory Virus Season https://emergency.cdc.gov/han/2023/han00499.asp (2023).

[32] Centers for Disease Control and Prevention. Updated guidance for healthcare providers on increased supply of nirsevimab to protect young children from severe Respiratory Syncytial Virus (RSV) during the 2023–2024 respiratory virus season https://emergency.cdc.gov/newsletters/coca/2024/010524a.html (2023).

[33] Jenco, M. Sanofi raising price of RSV immunization nirsevimab https://publications.aap.org/aapnews/news/28657/Sanofi-raising-price-of-RSV-immunization?autologincheck=redirected (2024).

[34] National Advisory Committee on Immunization. Statement on the prevention of respiratory syncytial virus (RSV) disease in infants www.canada.ca/content/dam/phac-aspc/documents/services/publications/vaccines-immunization/national-advisory-committee-immunization-statement-prevention-respiratory-syncytial-virus-disease-infants/naci-statement-2024-05-17.pdf (2024).

[35] De Luca, D., Sanchez-Luna, M., Schettler, K., Bont, L. & Baraldi, E. Universal infant immunisation against respiratory syncytial virus and European inequalities: the pandemics lesson has not been learnt. *Lancet Reg. Health Eur.* **34**, 100753 (2023). 37927432 10.1016/j.lanepe.2023.100753 PMC10624979

[36] Neumann, S. & Alverson, B. Nirsevimab: the hidden costs. *Hosp. Pediatr.* **14**, e2024007739 (2024). 38721666 10.1542/hpeds.2024-007739

[37] CDC. Immunizations to protect infants. Respiratory Syncytial Virus Infection (RSV) https://www.cdc.gov/rsv/vaccines/protect-infants.html (2024).

[38] Hak, S. F., Venekamp, R. P., Wildenbeest, J. G. & Bont, L. J. Outpatient respiratory syncytial virus infections and novel preventive interventions. *Curr. Opin. Pediatr.* **36**, 171–181 (2024). 38085019 10.1097/MOP.0000000000001323 PMC10919273

[39] Kampmann, B. et al. Bivalent Prefusion F vaccine in pregnancy to prevent RSV illness in infants. *N. Engl. J. Med.* **388**, 1451–1464 (2023). 37018474 10.1056/NEJMoa2216480

[40] Morens, D. M., Taubenberger, J. K. & Fauci, A. S. Rethinking next-generation vaccines for coronaviruses, influenzaviruses, and other respiratory viruses. *Cell Host Microbe* **31**, 146–157 (2023). 36634620 10.1016/j.chom.2022.11.016 PMC9832587

[41] Ison, M. G. et al. Efficacy and safety of respiratory syncytial virus prefusion F protein vaccine (RSVPreF3 OA) in older adults over 2 RSV seasons. *Clin. Infect. Dis.* **78**, 1732–1744 (2024). 38253338 10.1093/cid/ciae010 PMC11175669

[42] Friedland, L. GSK’s RSVPreF3 OA Vaccine (AREXVY). US Centers for Disease Control and Prevention www.cdc.gov/vaccines/acip/meetings/downloads/slides-2023-06-21-23/03-RSV-Adults-Friedland-508.pdf (2023).

[43] Gurtman, A. RSVpreF older adults: clinical development program updates. US Centers for Disease Control and Prevention https://stacks.cdc.gov/view/cdc/129992 (2023).

[44] Li, X. et al. Cost-effectiveness of respiratory syncytial virus preventive interventions in children: a model comparison study. *Value Health* **26**, 508–518 (2023). 36442831 10.1016/j.jval.2022.11.014

[45] Black, C. L. et al. Influenza, updated COVID-19, and respiratory syncytial virus vaccination coverage among Adults - United States, Fall 2023. *MMWR Morb. Mortal. Wkly Rep.* **72**, 1377–1382 (2023). 38127675 10.15585/mmwr.mm7251a4 PMC10754266

[46] The Lancet Respiratory Medicine. Respiratory syncytial virus vaccines: the future is bright. *Lancet Respir. Med.* **12**, 499 (2024). 38851193 10.1016/S2213-2600(24)00184-X

[47] CDC. RSVVaxView. https://www.cdc.gov/rsvvaxview/dashboard/index.html (2024).

[48] Public Health Agency of Canada. Highlights from the 2023-2024 seasonal influenza (flu) vaccination coverage survey www.canada.ca/en/public-health/services/immunization-vaccines/vaccination-coverage/seasonal-influenza-survey-results-2023-2024.html (2024).

[49] IDM. Welcome to SynthPops — SynthPops 1.10.4 documentation https://docs.idmod.org/projects/synthpops/en/latest/ (accessed 3 December 2024)

[50] Kerr, C. C. et al. Covasim: an agent-based model of COVID-19 dynamics and interventions. *PLoS Comput. Biol.* **17**, e1009149 (2021). 34310589 10.1371/journal.pcbi.1009149 PMC8341708

[51] Walsh, E. E. et al. Efficacy and safety of a bivalent RSV Prefusion F vaccine in older adults. *N. Engl. J. Med.* **388**, 1465–1477 (2023). 37018468 10.1056/NEJMoa2213836

[52] Schmoele-Thoma, B. et al. Vaccine efficacy in adults in a respiratory syncytial virus challenge study. *N. Engl. J. Med.* **386**, 2377–2386 (2022). 35731653 10.1056/NEJMoa2116154

[53] Heikkinen, T., Valkonen, H., Waris, M. & Ruuskanen, O. Transmission of respiratory syncytial virus infection within families. *Open Forum Infect. Dis.* **2**, ofu118 (2015). 25884006 10.1093/ofid/ofu118 PMC4396434

[54] Yamin, D. et al. Vaccination strategies against respiratory syncytial virus. *Proc. Natl Acad. Sci. USA* **113**, 13239–13244 (2016). 27799521 10.1073/pnas.1522597113 PMC5135296

[55] Hodgson, D. et al. Estimates for quality of life loss due to Respiratory Syncytial Virus. *Influenza Other Respir. Viruses* **14**, 19–27 (2020). 31625688 10.1111/irv.12686 PMC6928035

[56] Vega, T. et al. Influenza surveillance in Europe: comparing intensity levels calculated using the moving epidemic method. *Influenza Other Respir. Viruses* **9**, 234–246 (2015). 26031655 10.1111/irv.12330 PMC4548993

[57] Vynnycky, E. & White, R. *An Introduction to Infectious Disease Modelling* (Oxford Univ. Press, 2010).

[58] Spencer, J. A. et al. Distinguishing viruses responsible for influenza-like illness. *J. Theor. Biol.* **545**, 111145 (2022). 35490763 10.1016/j.jtbi.2022.111145

[59] Du, Z. et al. Comparative cost-effectiveness of SARS-CoV-2 testing strategies in the USA: a modelling study. *Lancet Public Health* **6**, e184–e191 (2021). 33549196 10.1016/S2468-2667(21)00002-5 PMC7862022

[60] Prem, K., Cook, A. R. & Jit, M. Projecting social contact matrices in 152 countries using contact surveys and demographic data. *PLoS Comput. Biol.* **13**, e1005697 (2017). 28898249 10.1371/journal.pcbi.1005697 PMC5609774

