# Supplementary material for: Impact of RSVpreF vaccination on reducing the burden of respiratory syncytial virus in infants and older adults
Source: Nat Med. 2025 Jan 9;31(2):647–52. doi: 10.1038/s41591-024-03431-7 (PMC11835734; doi:10.1038/s41591-024-03431-7)
Supplement: Supplementary file 1 — Supplementary Files 1 and 2. [file 41591_2024_3431_MOESM1_ESM.pdf]

nature medicine

Supplementary information https://doi.org/10.1038/s41591-024-03431-7

Impact of RSVpreF vaccination on reducing the burden of respiratory syncytial virus in infants and older adults

In the format provided by the authors and unedited

Supplementary File 1

Impact of RSVpreF Vaccination on Reducing the Burden of Respiratory Syncytial Virus in Infants and Older Adults

Zhanwei Du, Abhishek Pandey, Seyed M. Moghadas, Yuan Bai, Lin Wang, Laura Matrajt, Burton H. Singer, Alison P. Galvani

This Supplementary material provides additional details of the model structure, data sources, and parameters used for model calibration and simulations of vaccination scenarios.

Model Structure

We constructed the individual-based network incorporating community-specific and age-stratified heterogeneities using demographics of each country studied here (**Table S1**). Represented by the schematic diagram in **Figure S3**, infants (S_{infant}) are considered to be immunologically naïve to RSV infection without prior exposure. If infected, infants may develop symptomatic disease (Y_{infant}) or remain asymptomatic (A_{infant}). Individuals older than one year (S) were assumed to have a reduced susceptibility as a result of prior exposure to RSV. The level of susceptibility for each individual was sampled from Beta distributions corresponding to secondary or subsequent infections (**Table S4**). Similar to infection in infants, infected individuals at any age may develop symptomatic (Y) or asymptomatic (A) infection. Pregnant women and adults aged 60 years or older may be vaccinated against RSV disease, which reduces the risk of severe outcomes and hospitalization (H). Protection level of vaccinated individuals ($V_{o/m}$) was sampled from Beta distributions, reflecting the estimated ranges of vaccine efficacy in clinical trials for preventing severe RSV LRTI, used against hospitalization in our model (**Table S9**). Infants whose mothers were vaccinated with RSVpreF vaccine (V_{infant}), were assigned a sampled level of protection against hospitalization (from the corresponding Beta distributions) reflecting the estimates from clinical trials for maternal immunization (**Table S9**). Recovered individuals (R) are assumed to have a transient naturally-acquired protection against RSV infection (**Table S4**).

We randomly initialized simulations for each scenario with a single infection at the start of RSV season in each country. For each scenario, 500 independent simulations were run with daily increments for a time horizon of one year.

Supplementary Tables

Table S1. Population structure of countries studied (year 2021).[1]

Country	Population size	% of the total population in different age groups					
		0–4 y	5–14 y	15–49 y	50–64 y	≥ 65 y	Birth cohort
U.S.	336,997,624	5.80	11.25	51.85	18.06	13.04	1.10
Germany	83,408,554	4.89	8.89	43.12	20.82	22.28	0.89
U.K.	67,281,039	5.92	11.12	49.41	16.72	16.83	1.12
France	64,531,444	5.30	11.38	43.82	18.18	21.32	0.99
Italy	59,240329	3.57	8.63	42.45	21.47	23.88	0.67
Canada	38,155012	4.83	10.71	46.13	18.94	19.39	0.91
Australia	25,921,089	6.30	12.22	48.31	16.27	16.90	1.22
The Netherlands	17,501,695	5.36	10.21	45.60	18.99	19.84	1.18
Sweden	10,467,097	5.43	11.36	45.79	16.82	20.60	1.02
Ireland	4,986,526	6.26	13.54	49.66	15.85	14.69	1.18
Israel	8,900,059	10.43	17.75	47.92	11.52	12.38	1.94
Spain	47,486,935	4.09	9.58	45.67	19.84	20.82	0.74
Japan	124,612,530	3.62	8.10	41.45	18.18	28.65	0.69

Table S2. Parameters for constructing contact networks (Supplementary File 2).

Table S3. Similarity of age-specific contact patterns between survey and our model. Estimated difference (and 95% Uncertainty Range) between real and simulated contact matrices (shown in Figure S1) using the Frobenius norm,[2] averaged over 500 simulated demographics..

Country	Household	School	Workplace
U.S.	0.0026 (0.0024, 0.0028)	0.0054 (0.0048, 0.0063)	0.0071 (0.0066, 0.0076)
Germany	0.0033 (0.0031, 0.0036)	0.0094 (0.0088, 0.0101)	0.0088 (0.0082, 0.0094)
U.K.	0.0025 (0.0024, 0.0027)	0.0085 (0.0078, 0.0093)	0.009 (0.0082, 0.0101)
France	0.0026 (0.0024, 0.0027)	0.005 (0.0042, 0.0062)	0.0073 (0.0068, 0.0078)

Italy	0.0035 (0.0034, 0.0037)	0.0077 (0.0067, 0.0087)	0.0085 (0.0081, 0.0089)
Canada	0.0028 (0.0027, 0.003)	0.0064 (0.0057, 0.0073)	0.0074 (0.0067, 0.0081)
Australia	0.0029 (0.0028, 0.003)	0.0055 (0.0052, 0.0063)	0.007 (0.0067, 0.008)
The Netherlands	0.0028 (0.0026, 0.003)	0.0116 (0.0114, 0.0119)	0.009 (0.0085, 0.0097)
Sweden	0.0029 (0.0028, 0.0031)	0.0054 (0.005, 0.0062)	0.007 (0.0066, 0.0075)
Ireland	0.0028 (0.0027, 0.003)	0.005 (0.0044, 0.006)	0.0069 (0.0064, 0.0076)
Israel	0.0026 (0.0024, 0.0027)	0.0045 (0.0038, 0.0056)	0.0068 (0.0065, 0.0074)
Spain	0.003 (0.0029, 0.0032)	0.0048 (0.0043, 0.0058)	0.0073 (0.0069, 0.0079)
Japan	0.0035 (0.0034, 0.0036)	0.0059 (0.0053, 0.0065)	0.0075 (0.0071, 0.008)

Table S4. Estimates of infection parameters and level of susceptibility.

**Parameters**	**Estimate**	**Source**
Average duration of naturally acquired protection	358.9 days	[3]
Average duration of symptomatic/asymptomatic infection for infants	6.16 days	[3]
Average duration of infection for others older than 1 years of age	5.36 days	[3]
Proportion of RSV asymptomatic infection	[9.1%, 17.3%, 52.1%, 76.3, 77.8%, 56%, 24%] for age groups [0, 1–4, 5–14, 15–39, 40–60, 61-70, ≥71]	[4,5]
Level of susceptibility to RSV infection	The susceptibility of individuals older than 1 year of age was assumed to be 0.89 (95% CrI: 0.85, 0.93) for a secondary infection compared to a primary infection, 0.81 (95% CrI: 0.74, 0.85) for a tertiary infection (if occurred) compared to secondary infection, and 0.33 (95% CrI: 0.31, 0.37) for a subsequent infection (if occurred) compared to a tertiary infection.<br>Beta distributions:<br>Beta(212.16, 26.22) for a secondary (relative to a primary) infection<br>Beta(149.31, 35.02) for a tertiary (relative to a secondary) infection	[6,7]

	Beta(306.37, 622.02) for a subsequent (relative to a tertiary) infection.	

Table S5. Annual incidence of hospitalizations. The probability of hospitalization per infection ($p^{a}_{infect,hosp}$) was estimated to be [0.0397, 0.0048, 0.0061, 0.0068, 0.0072, 0.000061, 0.001, 0.0097] for age groups [0, 1, 2, 3, 4, 5-44, 45-64, 65-100].[8] For each country, $p^{a}_{infect,hosp}$ for the older population was scaled to match the median estimate of the total hospitalizations reported for the 2018-19 RSV season without vaccination.

Country	Estimates	Note, source
U.S.	214 per 100,000 (adults aged ≥ 60 years)	The average annual incidence of hospitalizations is 214 per 100 000 adults aged 60 years or older in the United States, [9]
Germany	23744 (adults aged ≥ 65 years)	The average annual incidence of hospitalizations is 23744 (Range: 18521 – 28967) in the age group ≥65 years, [10]
U.K.	19473 (adults aged ≥ 65 years)	The average annual incidence of hospitalizations is 19473 (Range: 13922 – 25024) in age group ≥65 years, [10]
France	17807 (adults aged ≥ 65 years)	17807 (Range: 13901 – 21713) in adults aged ≥65 years, [10]
Italy	18553 (adults aged ≥ 65 years)	18553 (Range: 14388 – 22719) in adults aged ≥65 years, [10]
Canada	145.5 per 100,000 (adults aged ≥ 65 years)	The average annual incidence of hospitalization was 145.5 per 100,000 Canadian adults aged ≥50 years, [11]
Australia	64.4 per 100,000 (adults aged 65 – 74 years)<br>359.7 per 100,000 (adults aged ≥ 65 years)	The average annual incidence of hospitalizations was 464.2, 64.4, 359.7 per 100,000 population for age groups 0–4, 65–74, and ≥75 years, [12]
The Netherlands	3467 (adults aged ≥ 65 years)	3467 (Range: 2590 – 4343) in adults aged ≥65 years, [10]
Sweden	2561 (adults aged ≥ 65	2561 (Range: 1981 – 3143) in adults

	years)	aged ≥65 years, [10]
Ireland	1005 (adults aged ≥ 65 years)	1005 (Range: 831 – 1178) in adults aged ≥65 years, [10]
Israel	9 per 100,000 (adults aged ≥ 65 years)	9 per 100,000 adults aged ≥65 years, [13]
Spain	11135 (adults aged ≥ 65 years)	11135 (Range: 8405 – 13864) in adults aged ≥65 years, [10]
Japan	62627 (adults aged ≥ 60 years)	The total hospitalizations for Japan in 2019: 62627 (Range: 40600 – 96748) among adults aged ≥60 years, [14]

Table S6. Estimates of the effective reproduction number, R_e, informed by the weekly RSV positive rates in each country. We fitted the population-wide scaling factor ϕ to match R_e and its mean estimated from 500 independent stochastic simulations.

**Country**	R_e	**Year, Source**	**Fitted R_e in simulations**
U.S.	1.43	2019, [15]	1.45 (95% UR: 1.16 – 1.74)
Germany	1.99	2018–2019, [16]	1.98 (95% UR: 1.58 – 2.37)
U.K.	1.39	2018, [17]	1.39 (95% UR: 1.07 – 1.70)
France	1.64	2018, [16]	1.64 (95% UR: 1.29 – 1.98)
Italy	1.79	Assumed to be mean value of its neighboring countries (Germany, France, and Spain)	1.76 (95% UR: 1.46 – 2.07)
Canada	1.41	2018–2019, [16]	1.43 (95% UR: 1.15 – 1.72)
Australia	1.29	2018, [17]	1.31 (95% UR: 1.02 – 1.60)
The Netherlands	1.97	2018, [18]	1.88 (95% UR: 1.49 – 2.27)
Sweden	1.52	2018–2019, [16]	1.51 (95% UR: 1.20 – 1.82)

Ireland	1.47	2019, [16]	1.48 (95% UR: 1.29 – 1.67)
Israel	1.60	2018–2019, [16]	1.60 (95% UR: 1.26 – 1.94)
Spain	1.73	2019, [16]	1.69 (95% UR: 1.47 – 1.91)
Japan	1.46	2019, [19]	1.47 (95% UR: 1.17 – 1.76)

Table S7. Influenza vaccine coverage for pregnant women.

Country	Before the COVID-19 pandemic		During or After the COVID-19 pandemic	
	**Coverage**	**Year, Source**	**Coverage**	**Year, Source**
U.S.	53.7%	2019, [20]	38.1%	2023-2024, [21]
Germany	14.1%	2018-2019, [22]	17.5%	2021-2022, [23]
U.K.	45.0%	2019, [24]	32.1%	2023-2024, [25]
France	7.4%	2015-2016, [26]	21.1%	2019-2021, [27]
Italy	14.9%	2018–2019, [28]	38.0%	2022-2023, [29]
Canada	45.0%	2019, [30]	53.0%	2021, [30]
Australia	61.0%	2015, [31]	58.7%	2020, [32]
The Netherlands	29.0%	2021–2022, [33]	38.0%	Assumed to be the average of other countries
Sweden	29.0%	2018–2019, [34]	38.0%	
Ireland	61.7%	2017–2018, [34]	38.0%	
Israel	32.0%	2018, [24]	26.2%	2021, [35]
Spain	41.0%	2019, [24]	57.8%	2023-2024, [36]
Japan	47.4%	2018, [37]	38.0%	Assumed to be the average of other countries

Table S8. Influenza vaccine coverage for older adults.

**Country**	**Before the COVID-19 pandemic**		**During or After the COVID-19 pandemic**	
	**Coverage**	**Year, Source**	**Coverage**	**Year, Source**
U.S.	67.0%	2019, [38]	50.6%	2023–2024, [39]
Germany	38.8%	2019, [38]	43.3%	2021–2022, [23]
U.K.	72.4%	2019, [38]	77.8%	2023–2024, [25]
France	52.0%	2019, [38]	54.0%	2023–2024, [40]
Italy	53.1%	2019, [38]	56.7%	2022–2023, [41]
Canada	70.0%	2019, [24]	74.0%	2022–2023, [42]
Australia	56.2%	2019, [38]	64.0%	2023, [43]
The Netherlands	54.0%	2019, [24]	68.4%	2022–2023, [44]
Sweden	52.0%	2019, [24]	63.0%	2022–2023, [45]
Ireland	68.0%	2019, [24]	75.6%	2023–2024, [46]
Israel	59.8%	2019, [38]	58.6%	2022, [38]
Spain	54.7%	2019, [24]	66.0%	2023–2024, [36]
Japan	50.0%	2019, [38]	66.0%	2020, [38]

Table S9. Efficacy of RSVpreF (Abrysvo) vaccines against severe RSV LRTI in adults and through maternal immunization in infants. Beta distributions were used to sample vaccine efficacy for each vaccinated individual from the estimated 95% confidence interval. Parameters of a and b in $\mathrm{Beta}(a, b)$ were determined using the relation $b = a \times (1/\mathrm{VE} - 1)$, where VE is the mean vaccine efficacy. Parameterization was achieved by minimizing the mean squared error on the 95% CI of the vaccine efficacy.

**Population**	**Vaccine efficacy against severe RSV LRTI (used against hospitalization in the model)**	**Source**	**Beta distribution**
Older adult (aged ≥60 years) or pregnant women	88.9% (95.0% CI: 53.6%–98.7%)	[47–49]	Beta(4.49, 0.56)

Protection of infants with maternal immunization	67.7% (99.17% CI: 15.9%–89.5%) within 90 days after birth	[50]	Beta(3.95, 1.88), within 90 days after birth
	59.5% (99.17% CI: 8.3%–83.7%) within 120 days after birth		Beta(3.93, 2.68), within 120 days after birth
	56.4% (99.17% CI: 5.2%–81.5%) within 150 days after birth		Beta(3.93, 3.04), within 150 days after birth
	56.8% (99.17% CI: 10.1%–80.7%) within 180 days after birth		Beta(5.17, 3.93), within 180 days after birth
	33.3% (99.17% CI: 0%–62.9%) within 360 days after birth		Beta(4.85, 9.71), within 360 days after birth

Table S10. life expectancy (years) for age groups.

Country	Remaining years of life	Source
U.S.	[78.9, 78.3, …, 2.3] for [0, 1-4, …, ≥100]	[51]
Germany	[80, …, 1.95] for [0-4, …, ≥100]	[52]
U.K.	[80.95, 80.26, …, 2.24] for [0, 1-4, …, ≥100]	[53]
France	[82.18, 81.48, …, 2.24] for [0, 1-4, …, ≥100]	[54]
Italy	[82.97, 82.20, …, 6.21] for [0, 1-4, …, ≥100]	[55]
Canada	[81.97, …, 2.4] for [0, …, ≥100]	[56]
Australia	[83.25, 82.55, …, 3.25] for [0, 1-4, …, ≥100]	[57]
The Netherlands	[81.79, 81.08, …, 5.95] for [0, 1-4, …, ≥100]	[58]
Sweden	[82.40, 81.57, …, 5.77] for [0, 1-4, …, ≥100]	[59]
Ireland	[81.84, 81.09, …, 5.62] for [0, 1-4, …, ≥100]	[60]
Israel	[82.62, 81.87, …, 6.31] for [0, 1-4, …, ≥100]	[61]
Spain	[83.22, 82.44, …, 6.67] for [0, 1-4, …, ≥100]	[62]
Japan	[84.69, …, 5.26] for [0-4, …, ≥100]	[63]

Table S11. Estimates of death rate for hospitalized cases.

Country	Death rate	Source
U.S.	[0.10%, 0.00%, 0.00%, 2.40%, 8.68%] for age group [0–4, 5–17, 18–49, 50–59, ≥60] years	64
Germany	Assumed the same as Spain	
U.K.	Assumed the same as Spain	
France	6.6% for age group ≥60 years. Other groups are assumed to be the same as Spain	65
Italy	Assumed the same as Spain	
Canada	[7.6%, 8.1%, 14%] for age group [60–69, 70–79, ≥80] years[66]. Other age groups are similar to the US	66
Australia	4.7% for age group ≥65; 0.006% for children <16 years of age. Other age groups are assumed to be 0	67,68
The Netherlands	Assumed the same as Spain	
Sweden	Assumed the same as Spain	
Ireland	Assumed the same as Spain	
Israel	0.12% for infants <1 year of age and 6.4% for age group ≥65 years<br>6.4%for the age group 60–64. Other age groups are assumed to be the same as Spain	13
Spain	[0.09%, 2.77%, 4.20%, 4.37%, 5.92%, 6.31%, 8.49%, 11.95%] for age groups [0-1,30-39,40-49,50-59,60-69,70-79,80-89,>90] years. Other groups are assumed to be 0	69
Japan	5.56% for the age group ≥65 years; 5.56% for the age group of 60–64 years. Other groups are assumed to be the same as China [70] as 0.14%, and 0.00% for age groups 0-5 and 6-17, respectively.	67

Table S12. Hospitalization costs ($c_{H,a}$, US$).

Country	Costs estimate (2023 US$)	Source	Note
U.S.	[$10214, $12125, $19712, $20884, $17222] for age groups [<1, 1-59, 60-74, 75-84, ≥85] years	[71]	(1) full-term infants (0–11 months) with RSV [71]<br>(2) The mean direct medical costs per RSV hospitalization of adults in 18–49 years: $12,125 [72,73]. We assumed the age group (1–59 years) has the same costs<br>(3) The adjusted mean costs per RSV hospitalization for age groups 60–74 years, 74–84 years, and ≥85 years [73,74]
Germany	[$4614.5, $3615.7, $3921.5, $6910.2, $8221.4, $4965.4, $7947.5, $8929.8, $9493, $6718.8, $5161.2, $4429.7] for age groups [<1, 1-2, 3-9, 10-19, 20-29, 30-39, 40-49, 50-59, 60-69, 70-79, 80-89, ≥90] years	[75,76]	1 EUR = 1.1 USD<br>Hospitalization costs of those under 2 years of age [75]<br>Hospitalization costs of those older than 2 years[76]
U.K.	[$1397.3, $828.4] for age groups [<15, ≥15] years	[6]	1 GBP = 1.27 USD<br>Costs per RSV-related hospital admission of both short- and long-stay in England and Wales [6]
France	[$4370.3, $3573.2] for age groups [<1, ≥1] years		Children aged < 5 years with RSV<br>1 EUR = 1.1 USD<br>The median hospitalization costs for acute bronchiolitis in a French university medical center was estimated at €3,248.4 [77]<br>The mean hospitalization costs per hospitalized child in the first year of life was €3973 [78]
Italy	$6328.77 for all age groups	[79]	1 EUR = 1.1 USD

Canada	[$8473.5, $7142.3, $6632.3, $8975.3, $11242.5, $7005, $10655.3, $14839.5, $9789, $9795] for age groups [0, 1, 2–5, 6–17, 18–49, 50–59, 60–69, 70–79, 80–89, ≥90] years	[80,11]	1 CAD = 0.75 USD<br>For age groups less than 50 years[80]<br>For age groups over 50 years[11]
Australia	$17120 for all age groups	[81,82]	For age groups ≤5 years [83]
The Netherlands	$3421 for all age groups	[84]	1 EUR = 1.1 USD<br>Under than 1 year of age [85]
Sweden	$18755.39 for all age groups	[86]	1 SEK = 0.098 USD<br>Costs per RSV hospitalization of preterm infants in Sweden was 131997 SEK [86].<br>kr 131997 in 2005 equals to kr 191381.57 in 2023 [87]
Ireland	$3936.90 for all age groups	[88]	1 EUR = 1.1 USD
Israel	$3021 for all age groups	[13]	1 LTS = 0.27 USD<br>The average hospitalization costs per hospitalized child (0-4 years) is $3021. We assume the other age groups have the same hospitalization costs
Spain	[$2696, $2579.2, $2868.1, $2868.1] for age groups [<1, 1–2, 2–5, ≥6] years	[89]	1 EUR = 1.1 USD<br>€3362 in the first year of life (72.9% from hospitalizations), €3252 in the second (72.1%) year of life, and €3514 between 2 and 5 years old (74.2%)[89]
Japan	$3344 for all age groups	[90]	

Table S13. Simulated scenarios of vaccination uptake akin to seasonal influenza.

Scenario	Vaccine uptake	Comment
Baseline	As indicated in Tables S6 and S7	Main analysis with vaccination uptake rates prior to the COVID-19 pandemic
V1	Vaccination coverage of pregnant women: 62% in all countries<br>Vaccination coverage of older adults: 73% in all countries	Maximum uptake rates reported prior to the COVID-19 pandemic
V2	Vaccination coverage of pregnant women: 7% in all countries<br>Vaccination coverage of older adults: 38% in all countries	Minimum uptake rates reported prior to the COVID-19 pandemic
V3	As indicated in Tables S6 and S7	Uptake rates reported during or after the COVID-19 pandemic

Secondary Scenario Analyses

Table S14 (Scenario V1). Projected hospitalization, death, YLL and direct costs of hospitalizations averted per 100,000 population of older adults attributed to RSV vaccination with country-specific uptake rates (**Supplementary Table S13**) among adults aged 60 years or older.

Country	Estimate (95% Uncertainty Range)				
	**Hospitalization averted**	**Death averted**	**YLL averted**	**Costs averted, (× 1000 $US)**	**% of death averted**
US	140.29 (131.99 – 148.29)	12.18 (11.46 – 12.87)	183.80 (173.19 – 194.35)	2767 (2606 - 2927)	65.52 (63.28 – 68.02)
Germany	78.94 (76.48 – 81.71)	5.86 (5.64 – 6.08)	69.89 (67.66 – 72.08)	568 (550 - 585)	65.93 (64.28 – 67.62)
UK	99.47 (94.68 – 103.65)	7.34 (6.97 – 7.66)	92.72 (88.56 – 96.36)	82 (78 - 86)	65.21 (62.94 – 67.28)
France	79.21 (76.72 – 81.66)	5.23 (5.06 – 5.39)	80.74 (78.13 – 83.38)	283 (274 - 292)	65.58 (63.92 – 67.32)
Italy	76.01 (71.30 – 80.72)	5.74 (5.37 – 6.11)	75.08 (70.35 – 79.67)	481 (451 - 511)	65.49 (63.24 – 67.37)
Canada	140.81 (133.71 – 148.73)	13.60 (12.86 – 14.40)	168.82 (160.78 – 178.25)	1651 (1566 - 1742)	65.43 (63.14 – 67.61)
Australia	106.02 (98.63 – 112.45)	4.79 (4.45 – 5.10)	81.66 (75.79 – 87.25)	1815 (1689 - 1925)	65.64 (63.30 – 67.98)
The Netherlands	64.72 (62.92 – 66.63)	4.62 (4.48 – 4.77)	66.48 (64.58 – 68.42)	221 (215 - 228)	65.62 (64.02 – 67.48)
Sweden	63.98 (61.24 – 66.74)	4.92 (4.69 – 5.15)	57.92 (55.46 – 60.59)	1200 (1149 - 1252)	65.59 (63.26 – 67.59)
Ireland	72.51 (1.70 – 93.01)	5.08 (0.13 – 6.58)	72.30 (1.69 – 92.38)	285 (7 - 366)	65.22 (58.75 – 74.11)
Israel	18.22 (17.18 – 19.35)	1.17 (1.10 – 1.24)	26.05 (24.46 – 27.79)	55 (52 - 58)	65.27 (62.21 – 68.56)
Spain	65.52 (59.03 – 72.46)	4.88 (4.38 – 5.42)	68.36 (61.69 – 75.37)	188 (169 - 208)	65.56 (62.23 – 68.61)
Japan	102.96 (98.86 – 107.09)	5.72 (5.50 – 5.95)	85.90 (82.29 – 89.29)	344 (331 - 358)	65.67 (64.02 – 67.20)

Table S15 (Scenario V1). Projected hospitalization, death, YLL and direct costs of hospitalizations averted per 100,000 population of infants attributed to RSV vaccination with country-specific uptake rates (**Supplementary Table S13**) among pregnant women.

Country	Estimate (95% Uncertainty Range)				
	**Hospitalization averted**	**Death averted**	**YLL averted**	**Costs averted, (× 1000 $US)**	**% of death averted**
US	1099.33 (617.39 – 1421.69)	1.10 (0.62 – 1.42)	86.74 (48.71 – 112.17)	11229 (6306 - 14521)	45.71 (25.84 – 53.60)
Germany	1326.33 (599.70 – 1594.89)	1.19 (0.54 – 1.44)	95.50 (43.18 – 114.83)	6120 (2767 - 7360)	42.71 (19.45 – 49.19)
UK	1127.79 (561.98 – 1460.76)	1.01 (0.51 – 1.31)	82.17 (40.94 – 106.42)	1576 (785 - 2041)	41.01 (20.18 – 49.45)
France	1466.44 (682.04 – 1772.70)	1.32 (0.61 – 1.60)	108.46 (50.45 – 131.11)	6409 (2981 - 7747)	46.82 (21.62 – 53.31)
Italy	974.13	0.88	72.75	6165	47.33

	(533.95 – 1352.70)	(0.48 – 1.22)	(39.87 – 101.02)	(3379 - 8561)	(27.89 – 59.58)
Canada	1015.57 (559.00 – 1276.07)	1.02 (0.56 – 1.28)	83.25 (45.82 – 104.60)	8605 (4737 - 10813)	46.18 (27.12 – 55.55)
Australia	1060.67 (621.26 – 1385.56)	0.06 (0.04 – 0.08)	5.30 (3.10 – 6.92)	18159 (10636 - 23721)	45.94 (26.65 – 54.14)
The Netherlands	956.68 (583.72 – 1623.30)	0.86 (0.53 – 1.46)	70.42 (42.97 – 119.50)	3273 (1997 - 5553)	27.95 (17.70 – 46.96)
Sweden	1269.71 (613.23 – 1555.38)	1.14 (0.55 – 1.40)	94.17 (45.48 – 115.35)	23814 (11501 - 29172)	45.75 (22.40 – 52.51)
Ireland	241.15 (0.00 – 460.75)	0.22 (0.00 – 0.41)	17.76 (0.00 – 33.94)	949 (0 - 1814)	49.37 (23.53 – 71.97)
Israel	1347.51 (660.87 – 1546.84)	1.62 (0.79 – 1.86)	133.60 (65.52 – 153.36)	4071 (1996 - 4673)	45.57 (22.40 – 50.69)
Spain	569.79 (296.06 – 858.08)	0.51 (0.27 – 0.77)	42.68 (22.17 – 64.27)	1536 (798 - 2313)	47.47 (30.57 – 64.39)
Japan	1244.87 (571.20 – 1606.19)	1.74 (0.80 – 2.25)	147.60 (67.72 – 190.44)	4163 (1910 - 5371)	46.73 (22.37 – 55.10)

Table S16 (Scenario V2). Projected hospitalization, death, YLL and direct costs of hospitalizations averted per 100,000 population of older adults attributed to RSV vaccination with country-specific uptake rates (**Supplementary Table S13**) among adults aged 60 years or older.

**Country**	**Estimate (95% Uncertainty Range)**				
	**Hospitalization averted**	**Death averted**	**YLL averted**	**Costs averted, (× 1000 $US)**	**% of death averted**
US	72.93 (66.37 – 78.95)	6.33 (5.76 – 6.85)	95.66 (86.92 – 103.32)	1439 (1306 - 1559)	34.07 (31.48 – 36.49)
Germany	41.06 (38.53 – 43.20)	3.05 (2.86 – 3.22)	36.35 (33.95 – 38.39)	296 (277 - 310)	34.29 (32.25 – 36.08)
UK	51.88 (48.25 – 55.53)	3.82 (3.53 – 4.11)	48.24 (44.97 – 51.70)	43 (40 - 46)	34.01 (31.63 – 36.14)
France	41.20 (39.02 – 43.41)	2.72 (2.58 – 2.87)	42.04 (39.72 – 44.50)	147 (139 - 155)	34.09 (32.50 – 35.79)
Italy	39.52 (36.04 – 43.10)	2.98 (2.73 – 3.26)	39.05 (35.84 – 42.50)	250 (228 - 273)	34.03 (31.30 – 36.48)
Canada	73.52 (67.68 – 79.10)	7.08 (6.51 – 7.68)	87.98 (81.37 – 94.52)	861 (792 - 930)	34.08 (31.70 – 36.36)
Australia	55.02 (50.05 – 60.83)	2.49 (2.25 – 2.76)	42.36 (38.13 – 47.24)	942 (857 - 1041)	34.09 (31.33 – 36.82)
The Netherlands	33.68 (31.83 – 35.50)	2.40 (2.27 – 2.55)	34.54 (32.76 – 36.38)	115 (109 - 121)	34.13 (32.26 – 36.08)
Sweden	33.28 (30.90 – 35.51)	2.56 (2.37 – 2.75)	30.10 (27.83 – 32.21)	624 (580 - 666)	34.14 (31.93 – 36.29)
Ireland	37.43 (1.16 – 50.02)	2.64 (0.08 – 3.52)	37.20 (0.97 – 49.79)	147 (5 - 197)	33.71 (27.05 – 43.15)
Israel	9.47 (8.59 – 10.41)	0.61 (0.55 – 0.67)	13.57 (12.13 – 14.97)	29 (26 - 31)	33.98 (30.88 – 37.09)
Spain	34.04 (29.98 – 38.31)	2.54 (2.21 – 2.88)	35.53 (31.41 – 39.78)	98 (86 - 110)	34.14 (30.91 – 37.39)
Japan	53.50 (50.36 – 56.70)	2.97 (2.80 – 3.15)	44.65 (41.59 – 47.33)	179 (168 - 190)	34.08 (32.36 – 35.94)

Table S17 (Scenario V2). Projected hospitalization, death, YLL and direct costs of hospitalizations averted per 100,000 population of infants attributed to RSV vaccination with country-specific uptake rates (**Supplementary Table S13**) among pregnant women.

Country	Estimate (95% Uncertainty Range)				
	**Hospitalization averted**	**Death averted**	**YLL averted**	**Costs averted, (× 1000 $US)**	**% of death averted**
US	115.37 (25.05 – 205.80)	0.12 (0.03 – 0.21)	9.10 (1.98 – 16.24)	1178 (256 - 2102)	4.70 (1.06 – 8.23)
Germany	122.52 (26.28 – 201.38)	0.11 (0.02 – 0.18)	8.82 (1.89 – 14.50)	565 (121 - 929)	3.80 (0.82 – 6.41)
UK	126.91 (26.33 – 203.66)	0.11 (0.02 – 0.18)	9.25 (1.92 – 14.84)	177 (37 - 285)	4.39 (0.91 – 7.10)
France	152.05 (49.54 – 243.53)	0.14 (0.04 – 0.22)	11.25 (3.66 – 18.01)	665 (216 - 1064)	4.70 (1.56 – 7.37)
Italy	104.93 (0.00 – 229.80)	0.09 (0.00 – 0.21)	7.84 (0.00 – 17.16)	664 (0 - 1454)	5.37 (0.00 – 11.07)
Canada	102.21 (26.28 – 186.36)	0.10 (0.03 – 0.19)	8.38 (2.15 – 15.28)	866 (223 - 1579)	4.49 (1.09 – 8.45)
Australia	122.06 (25.95 – 210.16)	0.01 (0.00 – 0.01)	0.61 (0.13 – 1.05)	2090 (444 - 3598)	4.98 (1.13 – 8.65)
The Netherlands	121.44 (27.23 – 200.51)	0.11 (0.02 – 0.18)	8.94 (2.00 – 14.76)	415 (93 - 686)	3.62 (0.80 – 5.97)
Sweden	124.72 (30.53 – 212.93)	0.11 (0.03 – 0.19)	9.25 (2.26 – 15.79)	2339 (573 - 3994)	4.34 (1.09 – 7.34)
Ireland	26.78 (0.00 – 85.66)	0.02 (0.00 – 0.08)	1.97 (0.00 – 6.31)	105 (0 - 337)	5.03 (0.00 – 19.00)
Israel	137.98 (49.73 – 203.48)	0.17 (0.06 – 0.24)	13.68 (4.93 – 20.17)	417 (150 - 615)	4.58 (1.64 – 6.80)
Spain	46.30 (0.00 – 166.07)	0.04 (0.00 – 0.15)	3.47 (0.00 – 12.44)	125 (0 - 448)	4.16 (0.00 – 12.58)
Japan	136.52 (35.36 – 232.46)	0.19 (0.05 – 0.33)	16.19 (4.19 – 27.56)	457 (118 - 777)	4.93 (1.35 – 8.39)

Table S18 (Scenario V3). Projected hospitalization, death, YLL and direct costs of hospitalizations averted per 100,000 population of older adults attributed to RSV vaccination with country-specific uptake rates (**Supplementary Table S13**) among adults aged 60 years or older.

Country	Estimate (95% Uncertainty Range)				
	**Hospitalization averted**	**Death averted**	**YLL averted**	**Costs averted, (× 1000 $US)**	**% of death averted**
US	96.62 (89.91 – 104.95)	8.39 (7.80 – 9.11)	126.81 (117.62 – 137.41)	1907 (1777 - 2067)	45.39 (42.40 – 48.37)
Germany	46.78 (44.42 – 49.50)	3.47 (3.29 – 3.68)	41.40 (39.21 – 43.63)	337 (319 - 356)	39.11 (37.28 – 41.16)
UK	106.07 (101.66 – 109.91)	7.81 (7.48 – 8.13)	98.84 (94.75 – 102.21)	88 (84 - 91)	69.59 (67.53 – 71.36)
France	58.50 (56.22 – 61.01)	3.86 (3.71 – 4.03)	59.78 (57.26 – 62.53)	209 (201 - 218)	48.44 (46.72 – 50.71)
Italy	58.99 (54.77 – 63.24)	4.46 (4.13 – 4.79)	58.26 (54.08 – 62.50)	373 (347 - 400)	50.80 (48.73 – 53.15)

Canada	143.06 (135.26 – 150.62)	13.78 (13.01 – 14.60)	171.44 (162.60 – 180.67)	1681 (1588 - 1764)	66.52 (64.33 – 68.70)
Australia	92.73 (86.31 – 99.68)	4.20 (3.89 – 4.51)	71.60 (66.38 – 77.39)	1588 (1478 - 1707)	57.63 (54.72 – 59.93)
The Netherlands	60.67 (59.11 – 62.53)	4.33 (4.21 – 4.47)	62.28 (60.46 – 64.28)	208 (202 - 214)	61.51 (59.81 – 63.19)
Sweden	55.15 (52.34 – 57.91)	4.24 (4.01 – 4.45)	49.85 (47.60 – 52.31)	1034 (982 - 1086)	56.51 (54.08 – 58.76)
Ireland	75.45 (2.09 – 95.52)	5.29 (0.15 – 6.75)	75.22 (1.88 – 95.71)	297 (8 - 376)	67.47 (57.26 – 73.47)
Israel	14.61 (13.51 – 15.74)	0.94 (0.86 – 1.01)	20.92 (19.14 – 22.66)	44 (41 - 48)	52.29 (48.32 – 56.09)
Spain	59.40 (53.08 – 65.81)	4.42 (3.94 – 4.93)	61.89 (55.84 – 68.08)	170 (152 - 189)	59.35 (56.01 – 62.28)
Japan	92.90 (89.12 – 96.84)	5.17 (4.95 – 5.38)	77.43 (74.15 – 81.10)	311 (298 - 324)	59.16 (57.72 – 60.84)

Table S19 (Scenario V3). Projected hospitalization, death, YLL and direct costs of hospitalizations averted per 100,000 population of infants attributed to RSV vaccination with country-specific uptake rates (**Supplementary Table S13**) among pregnant women.

Country	Estimate (95% Uncertainty Range)				
	**Hospitalization averted**	**Death averted**	**YLL averted**	**Costs averted, (× 1000 $US)**	**% of death averted**
US	668.77 (335.39 – 888.05)	0.67 (0.34 – 0.89)	52.77 (26.46 – 70.07)	6831 (3426 - 9071)	27.43 (14.18 – 34.58)
Germany	308.78 (127.06 – 496.63)	0.28 (0.11 – 0.45)	22.23 (9.15 – 35.76)	1425 (586 - 2292)	9.83 (4.10 – 15.37)
UK	592.11 (277.72 – 801.81)	0.53 (0.25 – 0.72)	43.14 (20.23 – 58.42)	827 (388 - 1120)	21.17 (9.53 – 26.99)
France	393.36 (167.66 – 634.18)	0.35 (0.15 – 0.57)	29.09 (12.40 – 46.90)	1719 (733 - 2772)	12.22 (5.19 – 19.60)
Italy	576.90 (242.83 – 884.45)	0.52 (0.22 – 0.80)	43.08 (18.13 – 66.05)	3651 (1537 - 5598)	27.22 (12.99 – 39.66)
Canada	851.00 (455.17 – 1123.60)	0.85 (0.46 – 1.12)	69.76 (37.31 – 92.10)	7211 (3857 - 9521)	39.48 (20.95 – 48.62)
Australia	1018.54 (539.29 – 1314.81)	0.06 (0.03 – 0.08)	5.09 (2.69 – 6.57)	17437 (9233 - 22510)	44.11 (23.92 – 51.09)
The Netherlands	631.78 (319.39 – 1020.59)	0.57 (0.29 – 0.92)	46.51 (23.51 – 75.13)	2161 (1093 - 3491)	19.20 (9.54 – 30.02)
Sweden	784.11 (367.42 – 1007.21)	0.71 (0.33 – 0.91)	58.15 (27.25 – 74.70)	14706 (6891 - 18891)	27.82 (12.67 – 34.37)
Ireland	150.28 (0.00 – 326.36)	0.14 (0.00 – 0.29)	11.07 (0.00 – 24.04)	592 (0 - 1285)	30.70 (0.00 – 51.89)
Israel	541.20 (245.47 – 677.62)	0.65 (0.29 – 0.81)	53.66 (24.34 – 67.18)	1635 (742 - 2047)	18.25 (8.43 – 22.48)
Spain	523.88 (292.98 – 791.72)	0.47 (0.26 – 0.71)	39.24 (21.94 – 59.30)	1412 (790 - 2134)	44.21 (27.32 – 59.18)
Japan	744.02 (337.78 – 996.29)	1.04 (0.47 – 1.39)	88.22 (40.05 – 118.13)	2488 (1130 - 3332)	27.69 (12.75 – 35.03)

References

1. Population Division in Department of Economic and Social Affairs. World Population Prospects 2022. *United Nations* https://population.un.org/wpp/Download/Standard/Population/.
2. Golub, G. H. & Van Loan, C. F. *Matrix Computations*. (JHU Press, 1996).
3. Hodgson, D., Pebody, R., Panovska-Griffiths, J., Baguelin, M. & Atkins, K. E. Evaluating the next generation of RSV intervention strategies: a mathematical modelling study and cost-effectiveness analysis. *BMC Med.* **18**, 348 (2020).
4. Munywoki, P. K. *et al.* Frequent Asymptomatic Respiratory Syncytial Virus Infections During an Epidemic in a Rural Kenyan Household Cohort. *J. Infect. Dis.* **212**, 1711–1718 (2015).
5. Krauer, F. *et al.* Effectiveness and efficiency of immunisation strategies to prevent RSV among infants and older adults in Germany: a modelling study. *BMC Med.* **22**, 478 (2024).
6. Hodgson, D. *et al.* Protecting infants against RSV disease: an impact and cost-effectiveness comparison of long-acting monoclonal antibodies and maternal vaccination. *The Lancet Regional Health – Europe* **38**, 100829 (2024).
7. Hodgson, D. *et al.* Estimates for quality of life loss due to Respiratory Syncytial Virus. *Influenza Other Respi. Viruses* **14**, 19–27 (2020).
8. Koltai, M. *et al.* Determinants of RSV epidemiology following suppression through pandemic contact restrictions. *Epidemics* **40**, 100614 (2022).
9. Moghadas, S. M. *et al.* Cost-effectiveness of Prefusion F Protein-based Vaccines Against Respiratory Syncytial Virus Disease for Older Adults in the United States. *Clin. Infect. Dis.* (2023) doi:10.1093/cid/ciad658.
10. Osei-Yeboah, R. *et al.* Estimation of the Number of Respiratory Syncytial Virus-Associated Hospitalizations in Adults in the European Union. *J. Infect. Dis.* **228**, 1539–1548 (2023).

11. ElSherif, M. *et al.* Leveraging Influenza Virus Surveillance From 2012 to 2015 to Characterize the Burden of Respiratory Syncytial Virus Disease in Canadian Adults ≥50 Years of Age Hospitalized With Acute Respiratory Illness. *Open Forum Infect Dis* **10**, ofad315 (2023).
12. Nazareno, A. L. *et al.* Modelled estimates of hospitalisations attributable to respiratory syncytial virus and influenza in Australia, 2009-2017. *Influenza Other Respi. Viruses* **16**, 1082–1090 (2022).
13. Glatman-Freedman, A. *et al.* Respiratory Syncytial Virus hospitalization burden: a nation-wide population-based analysis, 2000-2017. *J. Infect.* **81**, 297–303 (2020).
14. Savic, M., Penders, Y., Shi, T., Branche, A. & Pirçon, J.-Y. Respiratory syncytial virus disease burden in adults aged 60 years and older in high-income countries: A systematic literature review and meta-analysis. *Influenza Other Respi. Viruses* **17**, e13031 (2023).
15. Respiratory syncytial virus laboratory data (NREVSS). *Tyler Data & Insights* https://healthdata.gov/dataset/Respiratory-Syncytial-Virus-Laboratory-Data-NREVSS/7zgq-bp9w/data.
16. Flunet. https://www.who.int/tools/flunet.
17. WHO - Flu RSV. https://ais.paho.org/phip/viz/ed_who_rsv.asp.
18. Influenza surveillance outputs. https://www.who.int/teams/global-influenza-programme/surveillance-and-monitoring/influenza-surveillance-outputs.
19. IDWR Comparison Graph with the Past 10 Years (Weekly Report) -RSV Infection. https://www.niid.go.jp/niid/en/10/2096-weeklygraph/1661-21rsv.html (2012).
20. Lindley, M. C. *et al.* Vital Signs: Burden and Prevention of Influenza and Pertussis Among Pregnant Women and Infants - United States. *MMWR Morb. Mortal. Wkly. Rep.* **68**, 885–892 (2019).
21. Influenza vaccination coverage among pregnant persons, United States. https://www.cdc.gov/flu/fluvaxview/dashboard/vaccination-coverage-pregnant.html

(2024).

22. Steffen, A., Rieck, T. & Siedler, A. Monitoring of Influenza Vaccination Coverage among Pregnant Women in Germany Based on Nationwide Outpatient Claims Data: Findings for Seasons 2014/15 to 2019/20. *Vaccines (Basel)* **9**, (2021).
23. Rieck T, Steffen A, Feig M, Siedler A: Impfquoten bei Erwachsenen in Deutschland – Aktuelles aus der KV-Impfsurveillance. Epidemiologisches Bulletin. **49**, 3–23 (2022).
24. Influenza vaccination coverage. https://immunizationdata.who.int/pages/coverage/flu.html.
25. Seasonal influenza vaccine uptake in GP patients in England: winter season 2023 to 2024. *GOV.UK* https://www.gov.uk/government/statistics/seasonal-influenza-vaccine-uptake-in-gp-patients-winter-season-2023-to-2024/seasonal-influenza-vaccine-uptake-in-gp-patients-in-england-winter-season-2023-to-2024.
26. Descamps, A., Launay, O., Bonnet, C. & Blondel, B. Seasonal influenza vaccine uptake and vaccine refusal among pregnant women in France: results from a national survey. *Hum. Vaccin. Immunother.* **16**, 1093–1100 (2020).
27. SPF. Couverture vaccinale contre la grippe des femmes enceintes, propositions de vaccination et étude des déterminants, France métropolitaine, 2019-2021. https://www.santepubliquefrance.fr/maladies-et-traumatismes/maladies-et-infections-respiratoires/grippe/documents/article/couverture-vaccinale-contre-la-grippe-des-femmes-enceintes-propositions-de-vaccination-et-etude-des-determinants-france-metropolitaine-2019-2021.
28. Vilca, L. M. *et al.* Differences between influenza and pertussis vaccination uptake in pregnancy: a multi-center survey study in Italy. *Eur. J. Public Health* **31**, 1150–1157 (2021).
29. Capodici, A. *et al.* Influenza Vaccine Uptake in Italy-The 2022-2023 Seasonal Influenza Vaccination Campaign in Italy: An Update from the OBVIOUS Project. *Vaccines (Basel)* **12**, (2024).

30. Results of the Survey on Vaccination during Pregnancy 2021. https://www.canada.ca/en/public-health/services/publications/vaccines-immunization/survey-vaccination-during-pregnancy-2021.html (2022).
31. Mak, D. B., Regan, A. K., Vo, D. T. & Effler, P. V. Antenatal influenza and pertussis vaccination in Western Australia: a cross-sectional survey of vaccine uptake and influencing factors. *BMC Pregnancy Childbirth* **18**, 416 (2018).
32. Homaira, N., He, W.-Q., McRae, J., Macartney, K. & Liu, B. Coverage and predictors of influenza and pertussis vaccination during pregnancy: a whole of population-based study. *Vaccine* **41**, 6522–6529 (2023).
33. Widdershoven, V., Reijs, R. P., Eskes, A., Verhaegh-Haasnoot, A. & Hoebe, C. J. P. A. Acceptance of vaccination against pertussis, COVID-19 and influenza during pregnancy: a cross-sectional study. *BMC Pregnancy Childbirth* **23**, 219 (2023).
34. Influenza in Sweden – Season 2018-2019. https://www.folkhalsomyndigheten.se/publikationer-och-material/publikationsarkiv/i/influenza-in-sweden-season-2018-2019/?pub=63511.
35. Influenza vaccination coverage. *Immunization Data* https://immunizationdata.who.int/global/wiise-detail-page/influenza-vaccination-coverage?CODE=ISR&YEAR=.
36. Vacunación antigripal: coberturas 2023-24. https://vacunasaep.org/profesionales/noticias/vacunacion-antigripal-coberturas-2023-24.
37. Shono, A., Hoshi, S.-L. & Kondo, M. Maternal influenza vaccination relates to receiving relevant information among pregnant women in Japan. *Hum. Vaccin. Immunother.* **16**, 1364–1370 (2020).
38. Health care use - Influenza vaccination rates - OECD Data. *theOECD* https://data.oecd.org/healthcare/influenza-vaccination-rates.htm.
39. Influenza vaccination coverage, adults 65 years and older, United States. https://www.cdc.gov/flu/fluvaxview/dashboard/vaccination-coverage-adults-65-over.htm

(2024).

40. Données de couverture vaccinale grippe par groupe d'âge. https://www.santepubliquefrance.fr/determinants-de-sante/vaccination/articles/donnees-de-couverture-vaccinale-grippe-par-groupe-d-age.
41. della Salute, M. Influenza. https://www.salute.gov.it/portale/influenza/dettaglioContenutiInfluenza.jsp?lingua=italiano&id=679&area=influenza&menu=vuoto.
42. Highlights from the 2022–2023 Seasonal Influenza (Flu) Vaccination Coverage Survey. https://www.canada.ca/en/public-health/services/immunization-vaccines/vaccination-coverage/seasonal-influenza-survey-results-2022-2023.html (2023).
43. Australian Government Department of Health & Care, A. Australian Influenza Surveillance Reports – 2023. *Australian Government Department of Health and Aged Care* https://www.health.gov.au/resources/collections/australian-influenza-surveillance-reports-2023?language=en (2024).
44. Vaccine Coverage Dutch National Influenza Prevention Program 2022: brief monitor. https://www.nivel.nl/nl/publicatie/vaccine-coverage-dutch-national-influenza-prevention-program-2022-brief-monitor.
45. Influenza in Sweden – Season 2022–2023. https://www.folkhalsomyndigheten.se/publikationer-och-material/publikationsarkiv/i/influenza-in-sweden-season-2022-2023/?pub=126761.
46. Vaccination - health protection surveillance centre. https://www.hpsc.ie/a-z/respiratory/influenza/seasonalinfluenza/vaccination/.
47. Walsh, E. E. *et al.* Efficacy and Safety of a Bivalent RSV Prefusion F Vaccine in Older Adults. *N. Engl. J. Med.* **388**, 1465–1477 (2023).
48. Gurtman, A. RSVpreF older adults: clinical development program updates. *US Centers for Disease Control and Prevention* https://www.cdc.gov/vaccines/acip/meetings/downloads/slides-2023-06-21-23/02-RSV-A

dults-Gurtman-508.pdf (2023).

49. Schmoele-Thoma, B. *et al.* Vaccine Efficacy in Adults in a Respiratory Syncytial Virus Challenge Study. *N. Engl. J. Med.* **386**, 2377–2386 (2022).
50. Kampmann, B. *et al.* Bivalent Prefusion F Vaccine in Pregnancy to Prevent RSV Illness in Infants. *N. Engl. J. Med.* **388**, 1451–1464 (2023).
51. Arias, E., Heron, M. & Xu, J. Q. United States life tables, 2014 National vital statistics report; vol 66 no 4. *Hyattsville, MD: National Center for Health Statistics* (2017).
52. Life expectancy in Germany by gender and age group 2018/2020. *Statista* https://www.statista.com/statistics/1127942/life-expectancy-average-gender-age-group-germany/.
53. Buxton, J. National life tables: UK. *Office for National Statistics* https://www.ons.gov.uk/peoplepopulationandcommunity/birthsdeathsandmarriages/lifeexpectancies/datasets/nationallifetablesunitedkingdomreferencetables (2021).
54. Life table. *Ined - Institut national d'études démographiques* https://www.ined.fr/en/everything_about_population/data/france/deaths-causes-mortality/mortality-tables/.
55. GHO | By category | Life tables by country - Italy.
56. Government of Canada & Canada, S. Life expectancy and other elements of the complete life table, three-year estimates, Canada, all provinces except Prince Edward Island. Government of Canada, Statistics Canada (2022).
57. Deaths in Australia. *Australian Institute of Health and Welfare* https://www.aihw.gov.au/reports/life-expectancy-death/deaths-in-australia/contents/life-expectancy.
58. GHO | By category | Life tables by country - Netherlands.
59. GHO | By category | Life tables by country - Sweden.
60. GHO | By category | Life tables by country - Ireland.
61. GHO | By category | Life tables by country - Israel.
62. GHO | By category | Life tables by country - Spain.

63. Abridged life tables for japan 2016. https://www.mhlw.go.jp/english/database/db-hw/lifetb20/index.html.

64. Van Effelterre, T. *et al.* Modeling Respiratory Syncytial Virus Adult Vaccination in the United States with a Dynamic Transmission Model. *Clin. Infect. Dis.* (2023) doi:10.1093/cid/ciad161.

65. Celante, H. *et al.* Prognosis of hospitalised adult patients with respiratory syncytial virus infection: a multicentre retrospective cohort study. *Clin. Microbiol. Infect.* **29**, 943.e1–943.e8 (2023).

66. Mac, S. *et al.* Burden of illness associated with Respiratory Syncytial Virus (RSV)-related hospitalizations among adults in Ontario, Canada: A retrospective population-based study. *Vaccine* **41**, 5141–5149 (2023).

67. Kurai, D. *et al.* Targeted Literature Review of the Burden of Respiratory Syncytial Infection among High-Risk and Elderly Patients in Asia Pacific Region. *Infect Dis Ther* **12**, 807–828 (2023).

68. Saravanos, G. L. *et al.* Respiratory Syncytial Virus-attributable Deaths in a Major Pediatric Hospital in New South Wales, Australia, 1998-2018. *Pediatr. Infect. Dis. J.* **41**, 186–191 (2022).

69. Heppe-Montero, M., Gil-Prieto, R., Del Diego Salas, J., Hernández-Barrera, V. & Gil-de-Miguel, Á. Impact of Respiratory Syncytial Virus and Influenza Virus Infection in the Adult Population in Spain between 2012 and 2020. *Int. J. Environ. Res. Public Health* **19**, (2022).

70. Luo, M. *et al.* Comparison of infections with respiratory syncytial virus between children and adults: a multicenter surveillance from 2015 to 2019 in Beijing, China. *Eur. J. Clin. Microbiol. Infect. Dis.* **41**, 1387–1397 (2022).

71. Bowser, D. M. *et al.* Cost of Respiratory Syncytial Virus Infections in US Infants: Systematic Literature Review and Analysis. *J. Infect. Dis.* **226**, S225–S235 (2022).

72. Choi, Y. *et al.* Cost determinants among adults hospitalized with respiratory syncytial virus in the United States, 2017-2019. *Influenza Other Respi. Viruses* **16**, 151–158

(2022).

73. Grace, M., Colosia, A., Wolowacz, S., Panozzo, C. & Ghaswalla, P. Economic burden of respiratory syncytial virus infection in adults: a systematic literature review. *J. Med. Econ.* **26**, 742–759 (2023).
74. Ackerson, B. *et al.* Cost of Hospitalization Associated With Respiratory Syncytial Virus Infection Versus Influenza Infection in Hospitalized Older Adults. *J. Infect. Dis.* **222**, 962–966 (2020).
75. Wick, M. *et al.* Inpatient burden of respiratory syncytial virus in children ≤2 years of age in Germany: A retrospective analysis of nationwide hospitalization data, 2019-2022. *Influenza Other Respi. Viruses* **17**, e13211 (2023).
76. Niekler, P., Goettler, D., Liese, J. G. & Streng, A. Hospitalizations due to respiratory syncytial virus (RSV) infections in Germany: a nationwide clinical and direct cost data analysis (2010–2019). *Infection* (2023) doi:10.1007/s15010-023-02122-8.
77. Dervaux, B., Van Berleere, M., Lenne, X., Wyckaert, M. & Dubos, F. Impact of RSV test positivity, patient characteristics, and treatment characteristics on the cost of hospitalization for acute bronchiolitis in a French university medical center (2010-2015). *Front Pediatr* **11**, 1126229 (2023).
78. Kramer, R., Duclos, A., VRS study group in Lyon, Lina, B. & Casalegno, J.-S. Cost and burden of RSV related hospitalisation from 2012 to 2017 in the first year of life in Lyon, France. *Vaccine* **36**, 6591–6593 (2018).
79. Bechini, A. *et al.* Costs and healthcare utilisation due to respiratory syncytial virus disease in paediatric patients in Italy: a systematic review. *Public Health* **227**, 103–111 (2023).
80. Rafferty, E. *et al.* Evaluating the Individual Healthcare Costs and Burden of Disease Associated with RSV Across Age Groups. *Pharmacoeconomics* **40**, 633–645 (2022).
81. Demont, C. *et al.* Economic and disease burden of RSV-associated hospitalizations in young children in France, from 2010 through 2018. *BMC Infect. Dis.* **21**, 730 (2021).
82. Consumer Price Index (CPI) - countries - list.

https://tradingeconomics.com/country-list/consumer-price-index-cpi?continent=america.

83. Brusco, N. K. *et al.* The 2018 annual cost burden for children under five years of age hospitalised with respiratory syncytial virus in Australia. *Commun. Dis. Intell.* **46**, (2022).

84. Marbus, S. D. *et al.* Incidence and costs of hospitalized adult influenza patients in The Netherlands: a retrospective observational study. *Eur. J. Health Econ.* **21**, 775–785 (2020).

85. Rietveld, E. *et al.* Anticipated costs of hospitalization for respiratory syncytial virus infection in young children at risk. *Pediatr. Infect. Dis. J.* **23**, 523–529 (2004).

86. Neovius, K., Buesch, K., Sandström, K. & Neovius, M. Cost-effectiveness analysis of palivizumab as respiratory syncytial virus prophylaxis in preterm infants in Sweden. *Acta Paediatr.* **100**, 1306–1314 (2011).

87. Sweden inflation calculator. https://www.in2013dollars.com/sweden/inflation/.

88. Patterson, K. & Redmond, S. The economic burden that Respiratory Syncytial Virus (RSV) imposes on Irish hospitals. https://www.ispor.org/docs/default-source/euro2023/isporeurope23pattersonee404poster133491-pdf.pdf?sfvrsn=16efdaf3_0.

89. Martinón-Torres, F. *et al.* Clinical and economic burden of respiratory syncytial virus in Spanish children: the BARI study. *BMC Infect. Dis.* **22**, 759 (2022).

90. Igarashi, A. *et al.* Inpatient and outpatient costs associated with respiratory syncytial virus in Japanese infants and older adults. *Future Virol.* **18**, 643–657 (2023).

Supplementary File 2

Table S2. Parameters for constructing contact networks.

Parameter	Country	Value	Year, Source
Contact patterns	All countries	See reference for details	2017,[1]
Population age distribution	U.S.	[0.0605, 0.0607, 0.0566, 0.0557, 0.0612, 0.0843, 0.0849, 0.0765, 0.0697, 0.0701, 0.0682, 0.0654, 0.0591, 0.0453, 0.0312, 0.0504] for [0-4y, 5-9y, 10-14y, 15-19y, 20-24y, 25-29y, 30-34y, 35-39y, 40-44y, 45-49y, 50-54y, 55-59y, 60-64y, 65-69y, 70-74y, >75y]	2017,[2]
	Germany	[0.0484, 0.0456, 0.0455, 0.0492, 0.0543, 0.0576, 0.065, 0.0648, 0.0604, 0.0619, 0.0797, 0.0812, 0.0695, 0.0576, 0.0458, 0.1135] for [0-4y, 5-9y, 10-14y, 15-19y, 20-24y, 25-29y, 30-34y, 35-39y, 40-44y, 45-49y, 50-54y, 55-59y, 60-64y, 65-69y, 70-74y, >75y]	2011,[3]
	U.K.	[0.062, 0.056, 0.058, 0.037, 0.094, 0.068, 0.066, 0.067, 0.073, 0.073, 0.064, 0.057, 0.060, 0.048, 0.039, 0.078] for [0-4y, 5-9y, 10-14y, 15-19y, 20-24y, 25-29y, 30-34y, 35-39y, 40-44y, 45-49y, 50-54y, 55-59y, 60-64y, 65-69y, 70-74y, >75y]	2018,[4,5]
	France	[0.055, 0.059, 0.061, 0.06, 0.057, 0.056, 0.06, 0.062, 0.06, 0.066, 0.067, 0.066, 0.061, 0.058, 0.055, 0.098] for [0-4y, 5-9y, 10-14y, 15-19y, 20-24y, 25-29y, 30-34y, 35-39y, 40-44y, 45-49y, 50-54y, 55-59y, 60-64y, 65-69y, 70-74y, >75y]	2021,[6]
	Italy	[0.0363, 0.0427, 0.0477, 0.0485, 0.0502, 0.0513, 0.0545, 0.0578, 0.0655, 0.0779, 0.0811, 0.0798, 0.0684, 0.0596, 0.0571, 0.0449, 0.0387, 0.0237, 0.0109, 0.0028, 0.0003] for [0-4y, 5-9y, 10-14y, 15-19y, 20-24y, 25-29y, 30-34y,	2019,[7]

		35-39y, 40-44y, 45-49y, 50-54y, 55-59y, 60-64y, 65-69y, 70-74y, 75-79y, 80-84y, 85-89y, 90-94y, 95-99y, 100+]	
	Canada	[0.0495, 0.0555, 0.0575, 0.0544, 0.0595, 0.0655, 0.0681, 0.0679, 0.0649, 0.0623, 0.0640, 0.0716, 0.0695, 0.0598, 0.0499, 0.0341, 0.0227, 0.0142, 0.0070, 0.0018, 0.0003] for [0-4y, 5-9y, 10-14y, 15-19y, 20-24y, 25-29y, 30-34y, 35-39y, 40-44y, 45-49y, 50-54y, 55-59y, 60-64y, 65-69y, 70-74y, 75-79y, 80-84y, 85-89y, 90-94y, 95-99y, 100+]	2021,[8]
	Australia	[0.066,0.064,0.064,0.06,0.061,0.068,0.073,0.072,0.064,0.065,0.061,0.06,0.056,0.049,0.043,0.031,0.021,0.013,0.006,0.002,0.001] for [0-4y,5-9y,10-14y,15-19y,20-24y,25-29y,30-34y,35-39y,40-44y,45-49y,50-54y,55-59y,60-64y,65-69y,70-74y,75-79y,80-84y,85-89y,90-94y,95-99y,≥100y]	2021,[6]
	The Netherland	[0.0493, 0.0509, 0.0543, 0.0579, 0.0642, 0.0646, 0.0654, 0.0610, 0.0594, 0.0620, 0.0729, 0.0719, 0.0659, 0.0579, 0.0535, 0.0406, 0.0261, 0.0149, 0.0060, 0.0014, 0.0001] for [0-4y, 5-9y, 10-14y, 15-19y, 20-24y, 25-29y, 30-34y, 35-39y, 40-44y, 45-49y, 50-54y, 55-59y, 60-64y, 65-69y, 70-74y, 75-79y, 80-84y, 85-89y, 90-94y, 95-99y, 100+]	2022,[9]
	Sweden	[0.0563, 0.0597, 0.0600, 0.0565, 0.0553, 0.0662, 0.0729, 0.0647, 0.0611, 0.0632, 0.0635, 0.0634, 0.0545, 0.0517, 0.0513, 0.0996] for [0-4y, 5-9y, 10-14y, 15-19y, 20-24y, 25-29y, 30-34y, 35-39y, 40-44y, 45-49y, 50-54y, 55-59y, 60-64y, 65-69y, 70-74y, 75-100y]	2022,[10]
	Ireland	[0.0635, 0.0726, 0.0722, 0.0632, 0.0587, 0.0562, 0.0630, 0.0780, 0.0801, 0.0745, 0.0632, 0.0585, 0.0506, 0.0468, 0.0399, 0.0271,	2023,[11]

		0.0178, 0.0093, 0.0037, 0.0011, 0.0001] for [0-4y, 5-9y, 10-14y, 15-19y, 20-24y, 25-29y, 30-34y, 35-39y, 40-44y, 45-49y, 50-54y, 55-59y, 60-64y, 65-69y, 70-74y, 75-79y, 80-84y, 85-89y, 90-94y, 95-99y, 100+]	
	Israel	[0.1010, 0.0952, 0.0854, 0.0790, 0.0718, 0.0681, 0.0661, 0.0642, 0.0618, 0.0562, 0.0473, 0.0438, 0.0414, 0.0392, 0.0316, 0.0480] for [0-4y, 5-9y, 10-14y, 15-19y, 20-24y, 25-29y, 30-34y, 35-39y, 40-44y, 45-49y, 50-54y, 55-59y, 60-64y, 65-69y, 70-74y, 75+]	2020,[12]
	Spain	[0.0370, 0.0450, 0.0520, 0.0540, 0.0520, 0.0530, 0.0570, 0.0620, 0.0760, 0.0840, 0.0800, 0.0750, 0.0670, 0.0560, 0.0480, 0.0410, 0.0280, 0.0210, 0.0100, 0.0030] for [0-4y, 5-9y, 10-14y, 15-19y, 20-24y, 25-29y, 30-34y, 35-39y, 40-44y, 45-49y, 50-54y, 55-59y, 60-64y, 65-69y, 70-74y, 75-79y, 80-84y, 85-89y, 90-94y, 95-99y]	2023,[13]
	Japan	[0.0367, 0.0413, 0.0434, 0.0456, 0.0481, 0.0490, 0.0526, 0.0593, 0.0673, 0.0783, 0.0693, 0.0630, 0.0592, 0.0655, 0.0731, 0.1481] for [0-4y, 5-9y, 10-14y, 15-19y, 20-24y, 25-29y, 30-34y, 35-39y, 40-44y, 45-49y, 50-54y, 55-59y, 60-64y, 65-69y, 70-74y, 75-100y]	2020,[14]
Household size distribution	U.S.	[0.2782, 0.3443, 0.1576, 0.1365, 0.0509, 0.0197, 0.0127] for the number of people in household [1, 2, 3, 4, 5, 6, >=7]	2019,[15]
	Germany	[0.423, 0.332, 0.119, 0.091, 0.035] for [1, 2, 3, 4, >5]	2019,[16]
	U.K.	[0.296, 0.341, 0.158, 0.142, 0.044, 0.014, 0.005] for [1, 2, 3, 4, 5, 6, >7]	2019,[17]
	France	[0.3821 0.3239 0.1301 0.1092 0.0391 0.0155] for [1,	

		2, 3, 4, 5, >6]	2021 [18]
	Italy	[0.3318, 0.2768, 0.1889, 0.1515, 0.0390, 0.0121] for the number of people in household [1, 2, 3, 4, 5, 6+]	2022,[19]
	Canada	[0.2935, 0.3421, 0.1465, 0.1337, 0.0842] for the number of people in household [1, 2, 3, 4, 5 or 5+]	2021,[8]
	Australia	[0.2560, 0.3350, 0.1590, 0.1540, 0.0630, 0.0330] for the number of people in household [1, 2, 3, 4, 5, 6+]	2021,[20]
	The Netherland	[0.3405, 0.3154, 0.1496, 0.1261, 0.0465, 0.0219] for the number of people in household [1, 2, 3, 4, 5, 6+]	2018,[21]
	Sweden	[0.4072, 0.3014, 0.1145, 0.1150, 0.0414, 0.0123, 0.0082] for the number of people in household [1, 2, 3, 4, 5, 6, 7]	2022,[22]
	Ireland	[0.2370, 0.2897, 0.1789, 0.1621, 0.0877, 0.0446] for the number of people in household [1, 2, 3, 4, 5, 6+]	2012,[23]
	Israel	[0.1988, 0.2488, 0.1408, 0.1602, 0.2514] for the number of people in household [1, 2, 3, 4, 5 or 5+]	2020,[12]
	Spain	[0.2319, 0.3009, 0.2166, 0.1854, 0.0474, 0.0177] for the number of people in household [1, 2, 3, 4, 5, 6+]	2013,[23]
	Japan	[0.3797, 0.2811, 0.1657, 0.1190, 0.0382, 0.0113, 0.0037, 0.0010, 0.0003, 0.0001] for the number of people in household [1, 2, 3, 4, 5, 6, 7, 8, 9, 10+]	2012,[14]
Household age distribution by family size	U.S.	[[1.0, 1.0, 1.0, 1.0, 1.0, 1.0, 1.0, 1.0, 1.0, 1.0, 1.0] for household head age brackets, [163.0, 999.0, 2316.0, 2230.0, 1880.0, 1856.0,	2019,[15]

		2390.0, 3118.0, 9528.0, 9345.0, 5584.0], [115.0, 757.0, 1545.0, 1907.0, 2066.0, 1811.0, 2028.0, 2175.0, 3311.0, 1587.0, 588.0], [135.0, 442.0, 1029.0, 1951.0, 2670.0, 2547.0, 2368.0, 1695.0, 1763.0, 520.0, 221.0], [61.0, 172.0, 394.0, 905.0, 1429.0, 1232.0, 969.0, 683.0, 623.0, 235.0, 94.0], [25.0, 81.0, 153.0, 352.0, 511.0, 459.0, 372.0, 280.0, 280.0, 113.0, 49.0], [24.0, 33.0, 63.0, 144.0, 279.0, 242.0, 219.0, 115.0, 157.0, 80.0, 16.0], [0.0, 0.0, 0.0, 0.0, 0.0, 0.0, 0.0, 0.0, 0.0, 0.0, 0.0]] for the number of people in household [1, 2, 3, 4, 5, 6, 7, >=8]	
	Germany	[[166430, 964350, 1252841, 998113, 772623, 974626, 1095094, 1004591, 921831, 834104, 836585, 1139029, 7557715] for household head age brackets, [28756, 307073, 657797, 628240, 506028, 695518, 933875, 1106365, 1305898, 1411332, 1345208, 1622420, 8659415], [5838, 72567, 255031, 451368, 519758, 720420, 838252, 757408, 578999, 381783, 213598, 184594, 3036001], [1769, 20015, 106531, 312364, 552643, 844759, 835960, 530494, 266038, 127783, 54471, 34123, 1861677], [566, 5288, 27083, 82522, 168207, 267221, 269760, 162030, 78007, 41280, 21479, 11475, 588105], [415, 3163, 12739, 32838, 66370, 109522, 116422, 72223, 41300, 30343, 20643, 10140, 294183]] for the number of people in household [1, 2, 3, 4, 5, >=6]	2014,[23]
	U.K.	[[474100, 723400, 814400, 1391300, 1536000, 3258000] for household head age brackets, [1090100, 1122100, 925700, 1780800, 2120200, 2569600], [466800, 1056800, 1025300, 1065900, 450900, 221000], [293000, 1124400, 1403500, 848400, 167000, 44900], [870406,	2019,[24]

		356400, 497549, 267143, 68165, 16133], [27711, 113400, 158311, 85000, 21689, 5133], [9897, 40500, 56540, 30357, 7746, 1833]] for the number of people in household [1, 2, 3, 4, 5, 6, >7]	
	France	[[557, 212201, 713468, 385240, 580449, 491863, 529733, 599860, 711573, 804592, 871710, 852241, 649211, 684769, 737246, 572106, 266727, 845528] for household head age brackets, [278, 32278, 314860, 512510, 479315, 336647, 361577, 493438, 758028, 1034157, 1207103, 1196033, 835540, 706657, 539383, 288834, 96084, 386896], [145, 6000, 74389, 248006, 485206, 441003, 475913, 560308, 572055, 416578, 254173, 154097, 82411, 59056, 42113, 20413, 7092, 27807], [60, 1770, 21503, 115111, 420202, 647072, 747218, 630025, 388430, 179844, 81300, 39708, 19175, 11541, 6908, 3203, 1153, 4430], [46, 726, 5471, 28428, 119716, 245798, 319238, 249439, 136209, 59060, 26451, 13140, 6229, 3356, 1777, 828, 305, 1159], [24, 605, 2651, 9974, 37992, 80794, 112626, 95107, 57895, 29898, 15782, 7794, 3915, 1733, 902, 351, 121, 500]] for the number of people in household [1, 2, 3, 4, 5, >=6]	2015,[25]
	Italy	[[1824, 9211, 134727, 341988, 495587, 570581, 563060, 550237, 498046, 472267, 525740, 532142, 676280, 730819, 747810, 553491, 200186, 56697, 6980], [1456, 1920, 40733, 175347, 328114, 367450, 350936, 366014, 377259, 478973, 730158, 800200, 919982, 773357, 566001, 290573, 79170, 17412, 1901], [57, 944, 25901, 118921, 330457, 506885, 537990, 572289, 560430, 572153, 563305, 390406, 307484, 201172, 124375, 58782, 16260, 4021, 484], [4, 243, 7804, 46842, 191390, 498244, 726420, 779944, 638604, 447529,	2012,[23]

		284226, 143675, 93837, 57841, 36103, 18167, 5157, 1236, 135], [5, 84, 2118, 11069, 41441, 110923, 184672, 210365, 173084, 118079, 74979, 42840, 33813, 25767, 18490, 9602, 2475, 489, 55], [2, 39, 1064, 5030, 14407, 31024, 49516, 58026, 49333, 37298, 29650, 21738, 19632, =15598, 10232, 4681, 1039, 256, 29]] for the number of people in household [1, 2, 3, 4, 5, 6+]	
	Canada	[[155700, 536620, 430545, 591220, 802150, 701620, 472555, 224365], [182605, 654510, 412850, 673485, 1158190, 1024595, 503420, 132495], [58275, 361320, 450410, 551595, 432655, 167490, 61485, 18530], [21240, 268020, 666725, 621925, 253400, 56935, 17145, 4845], [9745, 141665, 411540, 363765, 143715, 42115, 13630, 3205]] for the number of people in household [1, 2, 3, 4, 5]	2017,[26]
	Australia	[[0, 10068, 72145, 104068, 90093, 79295, 94253, 106912, 106125, 96886, 100966, 97532, 108013, 83253, 85452, 64647, 20660, 3541, 378], [0, 1539, 23950, 53685, 56607, 49603, 61094, 79923, 96736, 105028, 121537, 115099, 131243, 79927, 61624, 32333, 8766, 1276, 141], [0, 510, 8030, 27060, 45668, 53944, 70564, 85047, 80689, 61935, 45518, 28543, 24816, 13364, 8649, 3914, 996, 145, 15], [0, 128, 2139, 13076, 39484, 68134, 95425, 94718, 63032, 33778, 16707, 7460, 5209, 2319, 1393, 622, 159, 28, 2], [0, 34, 427, 3181, 12961, 25574, 35903, 35676, 23086, 12143, 5227, 1826, 1076, 476, 269, 121, 36, 8, 0], [0, 22, 182, 1050, 6541, 13484, 18584, 17864, 12700, 8472, 3094, 901, 410, 173, 99, 67, 21, 3, 0]] for the number of people in household [1, 2, 3, 4, 5, 6+]	2012,[23]

	The Netherland	[[2602, 8166, 69424, 128627, 122256, 107505, 107969, 116835, 124309, 128599, 129645, 102196, 109713, 124325, 120480, 81180, 23002, 4744, 455], [93, 1512, 33587, 94418, 81051, 64916, 70757, 89903, 124081, 166318, 197292, 160652, 150613, 128336, 83893, 37310, 7482, 1053, 76], [46, 399, 11129, 49148, 81729, 80410, 89086, 98789, 99290, 76539, 48320, 25294, 18999, 14630, 8996, 4247, 947, 172, 10], [27, 71, 3160, 21913, 72602, 109661, 122690, 119160, 79182, 36482, 15148, 6433, 4230, 3049, 1979, 974, 205, 48, 5], [19, 21, 770, 5499, 20293, 40519, 50558, 47995, 30197, 12368, 5226, 2440, 1794, 1168, 776, 436, 109, 15, 0], [79, 43, 352, 2342, 7533, 16131, 22447, 22003, 14378, 6917, 3736, 2101, 1660, 1031, 642, 450, 434, 951, 595]] for the number of people in household [1, 2, 3, 4, 5, 6+]	2015,[23]
	Sweden	[[0, 10817, 74292, 94505, 73271, 58942, 56285, 57115, 59115, 61590, 62706, 59788, 48142, 47555, 50745, 41702, 19555, 4215], [1, 1074, 16723, 36100, 35249, 29959, 32602, 36409, 44280, 64009, 85008, 87990, 59350, 43038, 31099, 16771, 4918, 622], [0, 122, 4219, 19057, 32093, 31656, 32327, 35950, 39673, 36248, 22951, 12308, 5756, 3640, 2564, 1435, 508, 102], [1, 10, 718, 8148, 28805, 52531, 62034, 54615, 39443, 21308, 8669, 3354, 1429, 955, 694, 417, 150, 35], [0, 0, 47, 1383, 7756, 22209, 31357, 27129, 16187, 7160, 2882, 1521, 951, 552, 418, 266, 112, 22], [0, 1, 6, 185, 1464, 4920, 8442, 7673, 4849, 2519, 1639, 1132, 734, 506, 382, 198, 68, 14]] for the number of people in household [1, 2, 3, 4, 5, 6+]	same as Norway 2014,[27]
	Ireland	[[159, 1923, 10932, 22180, 33076,	2012,[23]

		32177, 28886, 28553, 30685, 32819, 34315, 32579, 30587, 29842, 24178, 14001, 4304, 734, 70], [58, 2008, 20231, 53695, 58376, 34917, 24853, 24636, 29896, 39289, 48212, 46473, 37897, 28904, 18002, 8652, 2688, 442, 42], [31, 1180, 11189, 29684, 43140, 34057, 26733, 25906, 29351, 30228, 25047, 16224, 9941, 6850, 3970, 1857, 528, 87, 7], [11, 567, 4810, 15212, 32341, 46894, 46204, 41470, 33781, 22079, 12354, 6169, 3011, 1796, 905, 395, 116, 19, 2], [9, 183, 1504, 4950, 12464, 26159, 32084, 29005, 19843, 10157, 4799, 2132, 927, 539, 211, 106, 28, 2, 0], [8, 87, 687, 2226, 5498, 11660, 17037, 16055, 10592, 5263, 2404, 1087, 546, 314, 144, 55, 20, 4, 2]] for the number of people in household [1, 2, 3, 4, 5, 6+]	
	Israel	[[1013, 10535, 37289, 50001, 41734, 26618, 19877, 20142, 24071, 32569, 38712, 33872, 42493, 40427, 37406, 21803, 5971, 1428, 199], [571, 3538, 30120, 62483, 41226, 21291, 16431, 21478, 34047, 59896, 75545, 57409, 57509, 40184, 25576, 12680, 3734, 1028, 95], [210, 2149, 18188, 40769, 51173, 31104, 23554, 30264, 43087, 50300, 33756, 15398, 9532, 5276, 3179, 1428, 398, 174, 37], [169, 1304, 9243, 31619, 68103, 67159, 46841, 46990, 44680, 33063, 14207, 5992, 3179, 1459, 694, 405, 123, 23, 0], [75, 807, 3350, 16083, 35613, 57288, 56761, 49592, 32536, 16360, 6265, 2397, 1278, 709, 171, 114, 7, 7, 0], [105, 759, 1569, 9994, 32273, 51335, 56049, 46912, 26318, 12159, 5024, 2332, 1512, 783, 425, 66, 16, 34, 0]] for the number of people in household [1, 2, 3, 4, 5, 6+]	2022,[28]
	Spain	[[34, 2660, 17849, 43750, 58116, 55300, 46600, 48443, 54381,	2013,[23]

		62379, 70373, 76307, 89183, 99308, 81401, 45399, 12455, 2672, 217], [4, 1620, 18573, 64489, 83199, 64310, 52190, 60722, 85587, 124607, 158600, 164053, 154633, 127551, 75951, 32137, 7496, 1704, 132], [1, 1017, 11532, 43865, 107829, 128073, 112100, 114590, 117129, 104950, 81766, 54052, 38436, 26760, 14820, 6703, 1701, 414, 43], [0, 399, 3918, 15362, 52946, 111973, 131692, 126578, 91802, 54819, 31684, 18406, 12941, 9562, 5599, 2544, 671, 162, 8], [0, 136, 1106, 3936, 10826, 22580, 30301, 33055, 25548, 17275, 11677, 8253, 6761, 5544, 3275, 1363, 318, 70, 4], [0, 77, 518, 1540, 4107, 7524, 10593, 12476, 10939, 8854, 6845, 5575, 4758, 3647, 1980, 814, 171, 47, 1]] for the number of people in household [1, 2, 3, 4, 5, 6+]	
	Japan	[[128, 287623, 1690552, 1786904, 1257834, 1036189, 1029851, 1279028, 1283940, 1233765, 1145819, 1331095, 1578003, 1314415, 1158972, 1334321], [53, 5003, 128019, 463076, 542483, 514265, 606517, 903898, 1069103, 1266846, 1466918, 1903973, 2384549, 1904656, 1364436, 1056083], [24, 1451, 49724, 239194, 520296, 620391, 737899, 973014, 976922, 962999, 901753, 908092, 911164, 643774, 430327, 314825], [5, 225, 17195, 114159, 437982, 826452, 1085442, 1196927, 882309, 605412, 413212, 321654, 270592, 180140, 130351, 116716], [2, 41, 3087, 28310, 126615, 301185, 394428, 386033, 251559, 164317, 109480, 89954, 88047, 67879, 55644, 49048], [1, 11, 625, 5645, 24936, 61694, 84261, 85178, 58938, 42597, 38155, 50646, 66520, 50560, 34374, 22455], [0, 7, 201, 1676, 7351, 19234, 25603, 24765, 17603, 17721, 27341, 42446, 45424, 24733, 14869,	2020,[14]

		12088]] for the number of people in household [1, 2, 3, 4, 5, 6, 7+]	
School size distribution	U.S.	[0.0275, 0.0092, 0.2018, 0.3945, 0.1927, 0.0459, 0.0550, 0.3945, 0.1927, 0.0459, 0.0550, 0.0367, 0.0092, 0.0, 0.0275, 0.0, 0.0, 0.0] for the number of people in school [20-50, 51-100, 101-300, 301-500, 501-700, 701-900, 901-1100, 1101-1300, 1301-1500, 1501-1700, 1701-1900, 1901-2100, 2101-2300, >2301]	2019–2020,[2]
	Germany	NA	same as the U.K.
	U.K.	[0.01, 0.015, 0.444, 0.412, 0.036, 0.019, 0.052, 0.01, 0.001, 0, 0, 0, 0, 0] for the number of people in school [20-50, 51-100, 101-300, 301-500, 501-700, 701-900, 901-1100, 1101-1300, 1301-1500, 1501-1700, 1701-1900, 1901-2100, 2101-2300, >2301]	2019,[29]
	France	NA	same as the U.K.
	Italy	[0.4177] for the number of people in school [1,100]; [0.5811] for the number of people in school [100,1000]; [0.0013] for the number of people in school [1000+]	2020[30] 2021,[31]
	Canada	[0.6629] for the number of people in school [1,100]; [0.1378] for the number of people in school [100,1000]; [0.1993] for the number of people in school [1000+]	2021,[32] 2022,[33] 2019,[34]
	Australia	[0.6230] for the number of people in school [1,100]; [0.3050] for the number of people in school [100,1000]; [0.0720] for the number of people in school [1000+]	2023,[35]

	The Netherland	[0.160] for the number of people in school [1,100]; [0.772] for the number of people in school [100,1000]; [0.068] for the number of people in school [1000+]	2021,[36]
	Sweden	[0.6304] for the number of people in school [1,100]; [0.3648] for the number of people in school [100,1000]; [0.0048] for the number of people in school [1000+]	equal to Norway 2022, [37–39]
	Ireland	[0.0000] for the number of people in school [1,100]; [0.9940] for the number of people in school [100,1000]; [0.0060] for the number of people in school [1000+]	2020,[40]
	Israel	[0.0277] for the number of people in school [1,100]; [0.9675] for the number of people in school [100,1000]; [0.0048] for the number of people in school [1000+]	2020, [41,42]
	Spain	[0.0164] for the number of people in school [1,100]; [0.9806] for the number of people in school [100,1000]; [0.0029] for the number of people in school [1000+]	2022,[43]
	Japan	[0.0245] for the number of people in school [1,100]; [0.9620] for the number of people in school [100,1000]; [0.0134] for the number of people in school [1000+]	2022,[44]
Work size distribution	U.S.	[60050, 19002, 13625, 9462, 3190, 1802, 486, 157, 109] for the number of people in workplace [1-4, 5-9, 10-19, 20-49, 50-99, 100-249, 250-499, 500-999, >1000]	2019,[2]
	Germany	[0.83, 0.14, 0.03, 0.01, 1.00] for the number of people in workplace [1-9, 10-49, 50-249, >250]	2020,[23]

	U.K.	[0.789, 0.109, 0.054, 0.029, 0.01, 0.005, 0.002, 0.001, 0.001] for the number of people in workplace [1-4, 5-9, 10-19, 20-49, 50-99, 100-249, 250-499, 500-999, >1000]	2015,[45]
	France	[0.956, 0.024, 0.013, 0.006, 0.001] for the number of people in workplace [1-9, 10-19, 20-49, 50-249, >250]	2018,[46]
	Italy	[0.8604, 0.1214, 0.0160, 0.0027] for the number of people in workplace [1-9, 10-49, 50-249, 250+]	2021,[19]
	Canada	[0.0668, 0.1325, 0.1072, 0.0816, 0.1089, 0.0438, 0.4592] for the number of people in workplace [1-4, 5-19, 20-49, 50-99, 100-299, 300-499, 500]	2023,[47]
	Australia	[0.596, 0.287, 0.092, 0.023, 0.002] for the number of people in workplace [1-9, 10-19, 20-49, 50-249, 250+]	2023,[48]
	The Netherland	[0.9588, 0.0327, 0.0071, 0.0014] for the number of people in workplace [1-9, 10-49, 50-249, 250+]	2021,[49]
	Sweden	[0.966, 0.028, 0.005, 0.001] for the number of people in workplace [1-9, 10-49, 50-249, 250+]	2020,[50]
	Ireland	[0.6969, 0.0565, 0.0102, 0.2364] for the number of people in workplace [1-9, 10-49, 50-249, 250+]	2022,[51]
	Israel	[0.9220, 0.0620, 0.0140, 0.0020] for the number of people in workplace [1-9, 10-49, 50-249, 250]	2016,[52]
	Spain	[0.1228, 0.0887, 0.1538, 0.2418, 0.3929] for the number of people in workplace [1-9, 10-19, 20-49,	2021,[53]

		50-249, 250+]	
	Japan	[0.6117, 0.1876, 0.1106, 0.0368, 0.0269, 0.0158, 0.0065, 0.0018, 0.0013, 0.0007, 0.0003] for the number of people in workplace [1-4, 5-9, 10-19, 20-29, 30-49, 50-99, 100-199, 200-299, 300-499, 500-999, 1000+]	2019,[54]
Employment rate in workplaces	U.S.	[0.3, 0.693, 0.861, 0.838, 0.814, 0.761, 0.636, 0.294, 0.061] for [16-19y, 20-24y, 250-29y, 30-44y, 45-54y, 55-59y, 60-64y, 65-74y, >75y]	2019,[2]
	Germany	[0.437, 0.73] for [15-29y, 30-64y]	2022,[55]
	U.K.	[0.24, 0.24, 0.62, 0.62, 0.85, 0.71, 0.11] for [16y, 17y, 18-19y, 21-24y, 25-49y, 50-64y, >65y]	2022,[56,57]
	France	[0.128, 0.534, 0.774, 0.808, 0.820, 0.844, 0.840, 0.833, 0.751, 0.355, 0.086, 0.026, 0.012] for [15-19y, 20-24y, 25-29y, 30-34y, 35-39y, 40-44y, 45-49y, 50-54y, 55-59y, 60-64y, 65-69y, 70-74y, >75y]	2021,[56]
	Italy	[0.0649, 0.4450, 0.7040, 0.7942, 0.8089, 0.8032, 0.7883, 0.7489, 0.5971, 0.2463, 0.0952, 0.0478, 0.0032] for [15y-19y, 20y-24y, 25y-29y, 30y-34y, 35y-39y, 40y-44y, 45y-49y, 50y-54y, 55y-59y, 60y-64y, 65y-69y, 70y-74y, 75+]	2014,[12]
	Canada	[0.4814, 0.8039, 0.8819, 0.9161, 0.8966, 0.9033, 0.8530, 0.8549, 0.7149, 0.5406, 0.2801, 0.0711] for [15y-19y, 20y-24y, 25y-29y, 30y-34y, 35y-39y, 40y-44y, 45y-49y, 50y-54y, 55y-59y, 60y-64y, 65y-69y, 70+]	2023,[58]
	Australia	[0.467, 0.739, 0.801, 0.817, 0.833, 0.832, 0.829, 0.801, 0.734, 0.569] for [15y-19y, 20y-24y, 25y-29y, 30y-34y, 35y-39y, 40y-44y, 45y-49y, 50y-54y, 55y-59y,	2020,[56]

		60y-64y]	
	The Netherland	[0.649, 0.779, 0.87, 0.871, 0.86, 0.853, 0.871, 0.834, 0.794, 0.626, 0.207, 0.077, 0.02] for [15y-19y, 20y-24y, 25y-29y, 30y-34y, 35y-39y, 40y-44y, 45y-49y, 50y-54y, 55y-59y, 60y-64y, 65y-69y, 70y-74y, 75+]	2021,[57]
	Sweden	[0.215, 0.601, 0.804, 0.862, 0.88, 0.77, 0.192] for [15y-19y, 20y-24y, 25y-34y, 35y-44y, 45y-54y, 55y-64y, 65y-74y]	2021,[59]
	Ireland	[0.238, 0.63, 0.786, 0.82, 0.811, 0.818, 0.809, 0.769, 0.724, 0.547, 0.263, 0.136] for [15y-19y, 20y-24y, 25y-29y, 30y-34y, 35y-39y, 40y-44y, 45y-49y, 50y-54y, 55y-59y, 60y-64y, 65y-69y, 70y-74y]	2021,[57]
	Israel	[0.416, 0.808, 0.699] for [15y-24y, 25y-54y, 55y-64y]	2022,[57]
	Spain	[0.2298, 0.7717, 0.5767] for [15y-24y, 25y-54y, 55y-64y]	2022,[57]
	Japan	[0.4666, 0.8651, 0.7812] for [15y-24y, 25y-54y, 55+]	2022,[60]
Enrollment rate in schools	U.S.	[0.0, 0.529, 0.95, 0.987, 0.977, 0.793, 0.409, 0.113, 0.027, 0.0] for [0-2y, 3-4y, 5-9y, 10-14y, 15-17y, 18-19y, 20-24y, 25-34y, 35-50y, >51y]	2019-2020,[61]
	Germany	[0, 0.67, 0.94, 0.99, 0.87, 0.51, 0.2, 0.08, 0.03, 0.01] for [0-2y, 2y, 3-5y, 6-14y, 15-19y, 20-24y, 25-29y, 30-34y, 35-39y, 40-64y]	2019,[56]
	U.K.	[1, 1, 0.96, 0.97, 0.97, 0.97, 0.97, 0.97, 0.97, 0.97, 0.97, 0.97, 0.99, 0.97, 0.92, 0.69, 0.62, 0.57, 0.43, 0.29, 0.2, 0.16, 0.13, 0.11, 0.09, 0.08, 0.08, 0.06, 0.05, 0.02] for [3y, 4y, 5y, 6y, 7y, 8y, 9y, 10y, 11y, 12y, 13y, 14y, 15y, 16y, 17y, 18y,	2019,[56]

		19y, 20y, 21y, 22y, 23y, 24y, 25y, 26y, 27y, 28y, 29y, 30-34y, 35-39y, >40y]	
	France	[0, 1.000, 0.869, 0.380, 0.076, 0.023, 0.012, 0.004] for [1-2y, 3-14y, 15-19y, 20-24y, 25-29y, 30-34y, 35-39y, >40y]	2019,[56]
	Italy	[0.05, 0.15, 0.95, 0.99, 0.86, 0.37, 0.13, 0.05, 0.03, 0.01] for [0y-1y, 2y, 3y-5y, 6y-14y, 15y-19y, 20y-24y, 25y-29y, 30y-34y, 35y-39y, 40y-64y]	2020,[56]
	Canada	[0, 1, 0.97, 0.96, 0.92, 0.7, 0.65, 0.58, 0.54, 0.43, 0.35, 0.29, 0.17, 0.15, 0.11, 0.1, 0.09, 0.06, 0.04, 0.01] for [3y-5y, 6y-14y, 15y, 16y, 17y, 18y, 19y, 20y, 21y, 22y, 23y, 24y, 25y, 26y, 27y, 28y, 29y, 30y-34y, 35y-39y, 40y-64y]	2022,[62–64]
	Australia	[0.0, 0.62, 0.65, 0.82, 0.97, 1.0, 0.99, 1.0, 0.99, 0.93, 0.71, 0.68, 0.63, 0.54, 0.46, 0.39, 0.32, 0.28, 0.25, 0.22, 0.21, 0.16, 0.13, 0.06] for [0y-1y, 2y, 3y, 4y, 5y, 6y, 7y, 8y-15y, 16y, 17y, 18y, 19y-20y, 21y, 22y, 23y, 24y, 25y, 26y, 27y, 28y, 29y, 30y-34y, 35y-39y, 40y-64y]	2020,[56]
	The Netherland	[0, 0.89, 1, 0.93, 0.53, 0.17, 0.07, 0.04, 0.02] for [0y-2y, 3y-5y, 6y-14y, 15y-19y, 20y-24y, 25y-29y, 30y-34y, 35y-39y, 40y-64y]	2021,[57]
	Sweden	[0.4730, 0.9447, 0.9921, 0.8802, 0.43724, 0.2578, 0.1768, 0.1331, 0.0487] for [0y-2y, 3y-5y, 6y-14y, 15y-19y, 20y-24y, 25y-29y, 30y-34y, 35y-39y, 40y-64y]	2019,[56]
	Ireland	[0.25, 1, 0.94, 0.43, 0.12, 0.07, 0.05, 0.03] for [0y-2y, 3y-14y, 15y-19y, 20y-24y, 25y-29y, 30y-34y, 35y-39y, 40y-64y]	2019,[56]
	Israel	[0.57, 0.75, 1, 0.96, 0.67, 0.21, 0.19, 0.07, 0.04, 0.01] for [0y-1y,	2020,[56]

		2y, 3y-5y, 6y-14y, 15y-19y, 20y-24y, 25y-29y, 30y-34y, 35y-39y, 40y-64y]	
	Spain	[0.41, 0.63, 0.97, 0.98, 0.87, 0.46, 0.16, 0.07, 0.05, 0.01] for [0y-1y, 2y, 3y-5y, 6y-14y, 15y-19y, 20y-24y, 25y-29y, 30y-34y, 35y-39y, 40y-64y]	2020,[56]
	Japan	[0.03, 0.08, 0.95, 1.0, 0.99, 97.0, 0.806, 0.65] for [0y-1y, 2y, 3y-5y, 6y-15y, 16y, 17y, 18y-24y, 25y-34y]	2015,[65]

References

1. Prem, K., Cook, A. R. & Jit, M. Projecting social contact matrices in 152 countries using contact surveys and demographic data. *PLoS Comput. Biol.* **13**, e1005697 (2017).
2. U.S. Census Bureau. Explore Census Data. https://data.census.gov/cedsci/all?q=United%20States%20Census%20Bureau.
3. Population by age groups (from 2011). *Federal Statistical Office* https://www.destatis.de/EN/Themes/Society-Environment/Population/Current-Population/Tables/liste-agegroups.html.
4. UK population pyramid interactive. https://www.ons.gov.uk/peoplepopulationandcommunity/populationandmigration/populationestimates/articles/ukpopulationpyramidinteractive/2020-01-08 (2020).
5. Age groups. https://www.ethnicity-facts-figures.service.gov.uk/uk-population-by-ethnicity/demographics/age-groups/latest (2018).
6. Population Pyramids of the World from 1950 to 2100. *PopulationPyramid.net* https://www.populationpyramid.net/zh/%E6%BE%B3%E5%A4%A7%E5%88%A9%E4%BA%9A/2021/.
7. Italy: population by age group. *Statista* https://www.statista.com/statistics/789270/population-in-italy-by-age-group/.

8. Government of Canada & Canada, S. Profile table, Census profile, 2021 Census of Population - Canada [Country]. https://www12.statcan.gc.ca/census-recensement/2021/dp-pd/prof/details/page.cfm?Lang=E&DGUIDList=2021A000011124&GENDERList=1&STATISTICList=1&HEADERList=0&SearchText=Canada (2022).
9. Netherlands, S. Population pyramid. *Statistics Netherlands* https://www.cbs.nl/en-gb/visualisations/dashboard-population/population-pyramid.
10. Population by age and sex. Year 1860 - 2022. *Statistikdatabasen* https://www.statistikdatabasen.scb.se/pxweb/en/ssd/START__BE__BE0101__BE0101A/BefolkningR1860N/.
11. Population pyramids of the world from 1950 to 2100. *PopulationPyramid.net* https://www.populationpyramid.net/ireland/.
12. United Nations Statistics Division. Demographic and Social Statistics. https://unstats.un.org/unsd/demographic-social/products/dyb/.
13. Population pyramids of the world from 1950 to 2100. *PopulationPyramid.net* https://www.populationpyramid.net/spain/2023/.
14. Population Census 2020 Population Census Basic Complete Tabulation on Population and Households 2020Oct. *Portal Site of Official Statistics of Japan* https://www.e-stat.go.jp/en/stat-search/files?page=1&layout=datalist&toukei=00200521&tstat=000001136464&cycle=0&year=20200&month=24101210&tclass1=000001136466&tclass2val=0.
15. US Census Bureau. America’s Families and Living Arrangements: 2019. (2019).
16. Statistisches Bundesamt Deutschland - GENESIS-Online. (2022).
17. Guy, P. Families and households. Office for National Statistics (2022).
18. Household size. *Ined - Institut national d’études démographiques* https://www.ined.fr/en/everything_about_population/data/france/couples-households-families/households/.
19. OECD. Aspects of daily life - Household : Households size.

http://dati.istat.it/Index.aspx?QueryId=18306&lang=en.

20. Household size. https://profile.id.com.au/australia/household-size?WebID=10&BMID=50.
21. Household. https://population.un.org/Household/index.html.
22. Number and percentage of households by region, type of housing and size of household. Year 2012 - 2022. *Statistikdatabasen* https://www.statistikdatabasen.scb.se/pxweb/en/ssd/START__HE__HE0111__HE0111A/HushallT26/.
23. UNdata. http://data.un.org/Data.aspx?d=POP&f=tableCode:50.
24. Number of households by household size and age of household reference person (HRP), English regions and UK constituent countries, 2019. Office for National Statistics (2020).
25. UNdata. https://data.un.org/Data.aspx?d=POP&f=tableCode%3A326.
26. Government of Canada & Canada, S. Housing Indicators (5), Tenure Including Presence of Mortgage Payments and Subsidized Housing (7), Age of Primary Household Maintainer (9), Household Type Including Census Family Structure (9) and Household Size (8) for Owner and Tenant Households With Household Total Income Greater Than Zero in Non-farm, Non-reserve Private Dwellings of Canada, Provinces and Territories, Census Divisions and Census Subdivisions, 2016 Census - 25% Sample Data. https://www12.statcan.gc.ca/census-recensement/2016/dp-pd/dt-td/Rp-eng.cfm?TABID=2&LANG=E&A=R&APATH=3&DETAIL=0&DIM=0&FL=A&FREE=0&GC=01&GL=-1&GID=1257309&GK=1&GRP=1&O=D&PID=110575&PRID=10&PTYPE=109445&S=0&SHOWALL=0&SUB=0&Temporal=2017&THEME=121&VID=0&VNAMEE=&VNAMEF=&D1=4&D2=0&D3=1&D4=0&D5=0&D6=0 (2017).
27. UNdata. https://data.un.org/Data.aspx?q=household+size&d=POP&f=tableCode%3a50.
28. Households – Economic Characteristics and Housing Density, Based on Labour Force Survey, 2020.

https://www.cbs.gov.il/en/publications/Pages/2022/Households%E2%80%93Economic-Characteristics-and-Housing-Density-Based-on-Labour-Force-Survey-2020.aspx.

29. Uk, G. Schools, pupils and their characteristics. *Crown. Available online: https://explore-education-statistics. service. gov. uk/data-tables (accessed on 5 July 2020)* (2020).
30. Italy: number of public universities by region 2020. *Statista* https://www.statista.com/statistics/728414/number-of-public-universities-by-region-italy/.
31. Istat.It education and training. https://www.istat.it/en/education-and-training?data-and-indicators.
32. Government of Canada & Canada, S. Number of students enrolled in kindergarten. Government of Canada, Statistics Canada (2021).
33. Government of Canada & Canada, S. Postsecondary enrolments, by registration status, institution type, status of student in Canada and gender. Government of Canada, Statistics Canada (2022).
34. Council of Ministers of Education, Canada. *CMEC* https://www.cmec.ca/299/education-in-canada-an-overview/index.html.
35. Schools. *Australian Bureau of Statistics* https://www.abs.gov.au/statistics/people/education/schools/latest-release (2023).
36. CBS Statline. https://opendata.cbs.nl/#/CBS/en/dataset/03753eng/table?searchKeywords=school%20 size.
37. Short-Cycle Higher Education. in *The International Encyclopedia of Higher Education Systems and Institutions* 2534–2534 (Springer Netherlands, Dordrecht, 2020).
38. Children and kindergartens. https://www.udir.no/in-english/the-education-mirror-2022/kindergarten/children-and-kindergartens/.
39. Facts about education in Norway 2023. *SSB* https://www.ssb.no/en/utdanning/utdanningsniva/artikler/facts-about-education-in-norwa

y-2023 (2023).

40. Swagger UI. https://data.oireachtas.ie/.
41. Study in Israel: Education in Israel. *educations.com* https://www.educations.com/study-guides/asia/study-in-israel/education-system-17589.
42. Education- Statistical Abstract of Israel 2020 - No.71. https://www.cbs.gov.il/en/publications/Pages/2020/Education-Statistical-Abstract-of-Israel-2020-No-71.aspx.
43. Bienvenido a la Web del Ministerio de Educación y Formación Profesional. https://www.educacionyfp.gob.es.
44. School Basic Survey. *Ministry of Education, Culture, Sports, Science and Technology website* https://www.mext.go.jp/b_menu/toukei/chousa01/kihon/1267995.htm.
45. Nomis - nomis - official census and labour market statistics. https://www.nomisweb.co.uk/default.asp.
46. Gavilan Library Remote DB Access. https://www.statista.com/statistics/502717/number-of-enterprises-france-by-number-employees/.
47. Government of Canada & Canada, S. Employment for all employees by enterprise size, annual. Government of Canada, Statistics Canada (2023).
48. Counts of Australian Businesses, including Entries and Exits. *Australian Bureau of Statistics* https://www.abs.gov.au/statistics/economy/business-indicators/counts-australian-businesses-including-entries-and-exits/latest-release (2023).
49. Distribution of total enterprises in the Netherlands 2022, by size. *Statista* https://www.statista.com/statistics/870174/distribution-of-total-enterprises-in-the-netherlands-by-size/.
50. Structural business statistics. *Statistiska Centralbyrån* https://www.scb.se/en/finding-statistics/statistics-by-subject-area/business-activities/structure-of-the-business-sector/structural-business-statistics/.

51. Industry breakdown of companies in Ireland. https://www.hithorizons.com/eu/analyses/country-statistics/ireland.
52. SME and entrepreneurship performance in Israel. https://www.oecd-ilibrary.org/sites/9789264262324-6-en/index.html?itemId=/content/component/9789264262324-6-en.
53. Entrepreneurship - Employees by business size - OECD Data. *theOECD* https://data.oecd.org/entrepreneur/employees-by-business-size.htm.
54. Statistics Bureau, Ministry of Internal Affairs & Communications. Statistics Bureau home page/economic census for business frame. https://www.stat.go.jp/english/data/e-census/.
55. Labour market and education. http://www.instat.gov.al/en/themes/labour-market-and-education/.
56. OECD. Enrolment rates by age. https://stats.oecd.org/Index.aspx?DataSetCode=EAG_ENRL_RATE_AGE.
57. Employment - Employment rate by age group - OECD Data. *theOECD* https://data.oecd.org/emp/employment-rate-by-age-group.htm.
58. Government of Canada & Canada, S. Labour force characteristics by sex and detailed age group, monthly, unadjusted for seasonality (x 1,000). Government of Canada, Statistics Canada (2023).
59. Labour force surveys (LFS). *Statistiska Centralbyrån* https://www.scb.se/en/finding-statistics/statistics-by-subject-area/labour-market/labour-force-surveys/labour-force-surveys-lfs/.
60. Statistics Bureau, Ministry of Internal Affairs & Communications. Statistics Bureau home page/labour force survey/historical data. https://www.stat.go.jp/english/data/roudou/lngindex.html.
61. OSPI. Report Card Enrollment 2019-20 School Year. (2020).
62. Government of Canada & Canada, S. Age (in single years), average age and median age and gender: Canada, provinces and territories and economic regions. Government

of Canada, Statistics Canada (2022).

63. Government of Canada & Canada, S. Participation rate in education, population aged 15 to 29, by age and type of institution attended. Government of Canada, Statistics Canada (2022).

64. Government of Canada & Canada, S. Participation rate in education, population aged 18 to 34, by age group and type of institution attended. Government of Canada, Statistics Canada (2022).

65. Statistics Bureau, Ministry of Internal Affairs & Communications. Statistics Bureau home page/JAPAN STATISTICAL YEARBOOK 2015 - chapter 22 education. https://www.stat.go.jp/english/data/nenkan/back64/1431-22.html.
